# Foxp3-mediated blockage of ryanodine receptor 2 underlies contact-based suppression by regulatory T cells

**DOI:** 10.1172/JCI163470

**Published:** 2023-12-15

**Authors:** Xiaobo Wang, Shuang Geng, Junchen Meng, Ning Kang, Xinyi Liu, Yanni Xu, Huiyun Lyu, Ying Xu, Xun Xu, Xinrong Song, Bin Zhang, Xin Wang, Nuerdida Nuerbulati, Ze Zhang, Di Zhai, Xin Mao, Ruya Sun, Xiaoting Wang, Ruiwu Wang, Jie Guo, S.R. Wayne Chen, Xuyu Zhou, Tie Xia, Hai Qi, Xiaoyu Hu, Yan Shi

**Affiliations:** 1 Department of Basic Medical Sciences, School of Medicine, and; 2 Institute for Immunology, Beijing Key Lab for Immunological Research on Chronic Diseases, School of Medicine, Tsinghua University, Beijing, China.; 3 Department of Microbiology, Immunology and Infectious Diseases, Snyder Institute, University of Calgary, Calgary, Alberta, Canada.; 4 Peking University-Tsinghua University-National Institute of Biological Sciences Joint Graduate Program, School of Life Sciences, and; 5 Tsinghua-Peking Center for Life Sciences, Tsinghua University, Beijing, China.; 6 Department of Medical Oncology, Affiliated Hospital of Jiangnan University and Jiangsu Institute of Parasitic Diseases, Wuxi, Jiangsu, China.; 7 Libin Cardiovascular Institute, Department of Physiology and Pharmacology, University of Calgary, Calgary, Alberta, Canada.; 8 CAS Key Laboratory of Pathogenic Microbiology and Immunology, Institute of Microbiology, Chinese Academy of Sciences (CAS), Beijing, China.; 9 Collaborative Innovation Center for Biotherapy, Tsinghua University, Beijing, China.

**Keywords:** Autoimmunity, Immunology, Antigen-presenting cells, Tolerance

## Abstract

The suppression mechanism of Tregs remains an intensely investigated topic. As our focus has shifted toward a model centered on indirect inhibition of DCs, a universally applicable effector mechanism controlled by the transcription factor forkhead box P3 (Foxp3) expression has not been found. Here, we report that Foxp3 blocked the transcription of ER Ca^2+^-release channel ryanodine receptor 2 (RyR2). Reduced RyR2 shut down basal Ca^2+^ oscillation in Tregs, which reduced m-calpain activities that are needed for T cells to disengage from DCs, suggesting a persistent blockage of DC antigen presentation. RyR2 deficiency rendered the CD4^+^ T cell pool immune suppressive and caused it to behave in the same manner as Foxp3^+^ Tregs in viral infection, asthma, hypersensitivity, colitis, and tumor development. In the absence of Foxp3, *Ryr2*-deficient CD4^+^ T cells rescued the systemic autoimmunity associated with scurfy mice. Therefore, Foxp3-mediated Ca^2+^ signaling inhibition may be a central effector mechanism of Treg immune suppression.

## Graphical abstract

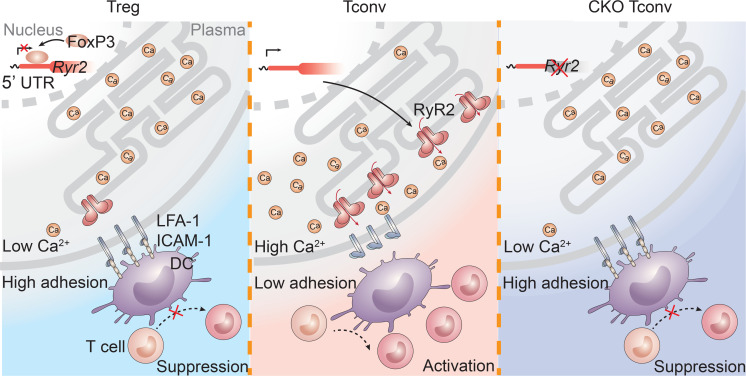

## Introduction

The suppressive mechanism of Tregs remains a topic of continuing debate. Proposed mechanisms, including T cell cytolysis, surface protein extraction, local generation of adenosine, etc., all require Treg binding to their targets of suppression (1). In more recent years, a string of reports has shifted our focus to the direct suppression of DCs. This focus has two natural rationales. On the one hand, Tregs are vastly outnumbered by CD4^+^ T cells, yet they retain a numerical advantage to DCs, with a rough ratio of 2 to 1 in vivo, inferring a more logical and judicious use of suppressive power. Second, the direct DC/Treg binding appears to be commonly observed both in vivo and in vitro (2–7). While such binding is regarded as exerting suppression on DCs, no consensus has been reached with regard to an exact mode of operation. Some of the reports suggested that this is mainly via the transendocytosis or trogocytosis of costimulatory molecules by CTLA-4 expressed on Tregs (8–12). Shevach’s group proposed that MHC class II extraction plays an important role in dampening DCs in antigen presentation (13). Our group believes that the binding between Tregs and DCs is mediated by LFA-1 and ICAM-1 and the binding itself is so exuberantly strong that DC cytoskeleton is too distorted to support antigen presentation to other T cells (2, 7). With time, these differences will likely be resolved to produce a more refined and comprehensive picture regarding how Tregs suppress DCs via contact. However, for the discussion to continue, two essential questions need to be answered: why do Tregs bind DCs with such a usually strong force and how does this excessive force may impact DCs?

Onishi et al. first used in vitro experiments to suggest that strong binding was mediated by LFA-1 (4). On that basis, we used single-cell force spectroscopy (SCFS) to provide a molecular explanation of how this may work. Tregs have intrinsically low m-calpain activities. The lack of this ubiquitous calcium-regulated protease renders Tregs unable to recycle their surface LFA-1, a basic mechanism used by CD4^+^ T cells to disengage from a transient binding partner (14–16); Tregs are, therefore, locked in a perpetual state of high adhesion. DCs, thus engaged, use their unique actin-bundling protein Fascin-1 to polarize their cortical cytoskeleton toward Treg binding sites, depriving them of their ability to form a stable contact with other T cells. As LFA-1 conformational changes and expression levels in Tregs are roughly equal to those of other T cells, low m-calpain activity becomes emblematic for Tregs (2). Whether this feature is essential to Treg suppression and how it is regulated by the master regulation of forkhead box P3 (Foxp3) are therefore interesting questions.

We report here that basal calcium oscillation in Tregs is severely depressed. mRNA expression analysis returned a list of calcium regulators with reduced abundance in Tregs. The most reduced is RyR2, a sarcoplasmic reticulum Ca^2+^ release channel with its cytoplasmic domain in close contact to the inner leaflet of the plasma membrane (17). Using several model cell lines, we found that Foxp3 expression autonomously suppressed *Ryr2* expression, targeting a stretch of guanosine-rich sequence roughly 200 bp before the *Ryr2* start codon. Conventional T cells (Tconvs) with *Ryr2* knockdown (KD) bound to DCs with forces similar to those of Tregs and became suppressive, echoing our early findings that m-calpain blockage imparts Treg-like inhibition to Tconvs. In addition, T cells with CD4^+^-specific *Ryr2* deletion became immune suppressive both in vitro and in vivo in the absence of Foxp3 expression. In the absence of Tregs, *Ryr2*^–/–^ Tconvs restored immune homeostasis in all aspects of immune deficiency in scurfy mice, reminiscent of Sakaguchi’s reports that cotransfer of untreated CD4^+^ T cells blocked the ability CD5^lo^CD4^+^ T cells to induce systemic autoimmunity in athymic mice (18) and that this suppressive capacity was associated with a subpopulation of CD25^+^ T cells (19). Our work, therefore, reveals a distinct, Foxp3-mediated effector function of Tregs and may provide the molecular base of the previously elusive contact-dependent suppression by Tregs.

## Results

### Reduced RyR2 activity is the basis of contact-dependent suppression by Tregs.

**Figure 1:**
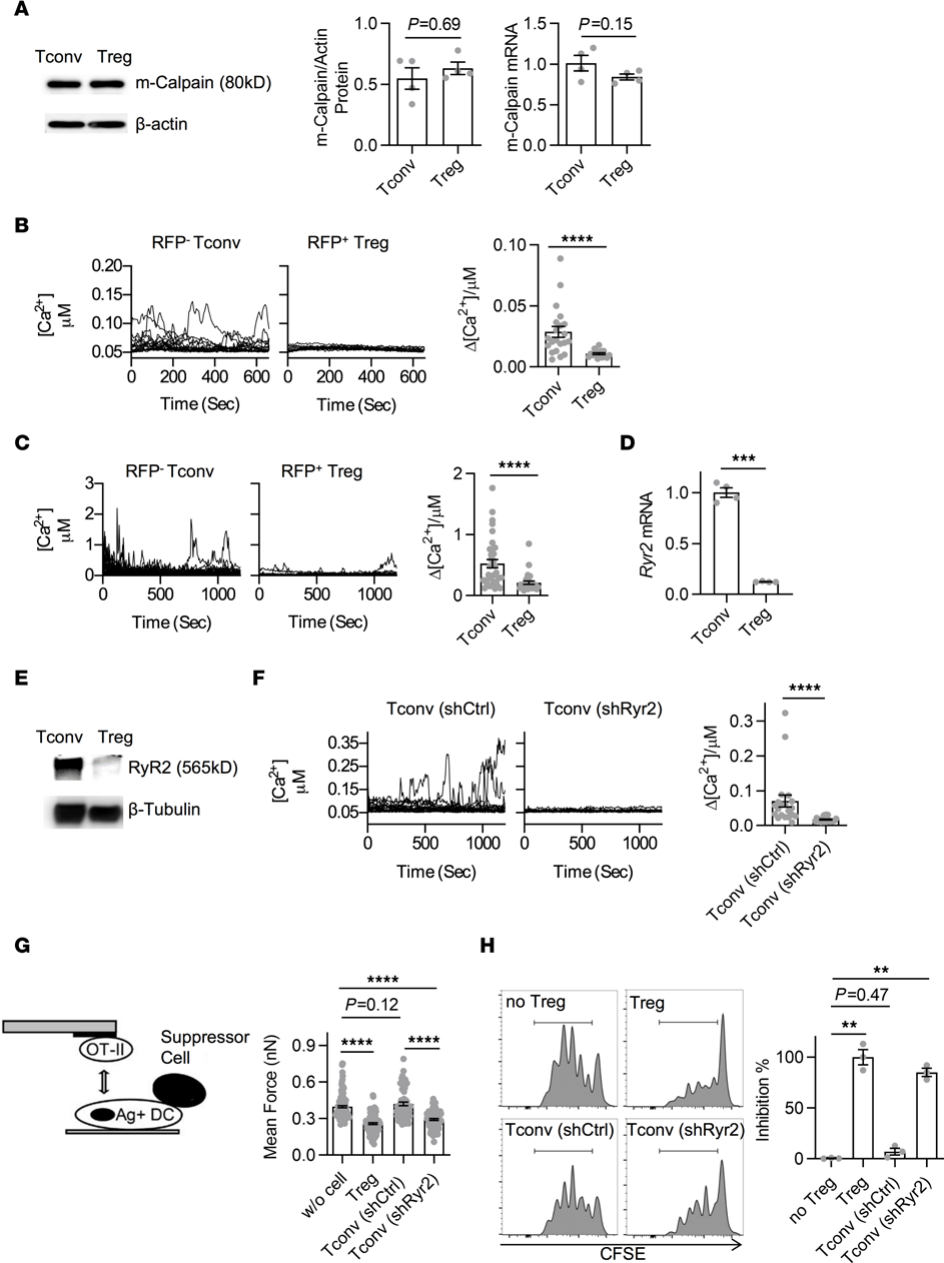
Reduced RyR2 activity is the basis of contact-dependent suppression. (**A**) Western blot protein (left) and qPCR RNA (right) analyses of m-calpain expression in Tconvs and Tregs isolated from Foxp3^GFP^ mice. Number of independent experiments [*N*] = 4. (**B**) The change of intracellular free Ca^2+^ concentration [Ca^2+^] over time (left) and corresponding amplitude (right) are shown. Resting RFP^–^ Tconvs and RFP^+^ Tregs were sorted from CD4^+^ splenocytes of FOXP3-IRES-RFP mice by FACS following CD4 immunomagnetic negative selection (MACS) and loaded with Fluo-4 AM with 1.2 mM Ca^2+^ after overnight culture. Each line represents 1 cell. Number of biological repeats in 1 group of data points [*n*] = 20, *N* = 5. (**C**) As in **B**, with ratiometric Ca^2+^ imaging using Cal Red R525/650. *n* = 20, *N* = 3. (**D**) qPCR analysis of *Ryr2* gene expression in FACS- or MACS-purified Tconvs and Tregs cultured overnight. *N* > 5. (**E**) Western blot analyses of RyR2 protein expression in overnight-cultured Tconvs and Tregs isolated from Foxp3^GFP^ or FOXP3-IRES-RFP mice by FACS. *N* = 3. (**F**) As in **B**, with the exception that shRNA-knockdown Tconvs were used in place of Tregs. *n* = 20, *N* = 3. (**G**) Adhesion between OT-II T cells and OVA-pulsed DC2.4 cells that were free or engaged by Tregs, *Ryr2* KD Tconvs, or control Tconvs on the opposite side of the DC cell bodies was analyzed. Shown are the triple-cell AFM assay setup and adhesion forces. *N* = 3. (**H**) DC occupation by Tregs, *Ryr2* KD Tconvs, or control KD Tcon-mediated suppression of OT-II T cell division. Left: FACS plots, normalized to mode. Right: Relative efficiency of inhibition using Tregs and no Tregs as 100% and 0%, respectively. *N* = 3. Two-tailed unpaired Student’s *t* test. **P < 0.01; ***P < 0.001; ****P < 0.0001. Data are shown as the mean ± SEM where applicable.

As m-calpain activities are reduced in Tregs (2), we analyzed whether its expression level is indeed low. As shown in Figure 1A, no difference in m-calpain was found between Tconvs and Tregs at the protein or mRNA level. Calpains are regulated by distinct intracellular Ca^2+^ availability. There was a persistent reduction of overall Ca^2+^ signal in Tregs (Supplemental Figure 1A; supplemental material available online with this article; https://doi.org/10.1172/JCI163470DS1). To study this in detail, we observed Ca^2+^ signals in both Tconvs and Tregs in resting state with two different indicators, Fluo-4 AM and ratiometric Cal Red R525/650 AM. While spontaneous Ca^2+^ oscillations are a common feature shared by many immune cells, including Tconvs, this activity was essentially absent in Tregs (Figure 1, B and C), supporting their low m-calpain activation.

Upon T cell activation, Ca^2+^ signal comes from IP3R Ca^2+^ release channels that expand secondary Ca2+ amplification events, such as the opening of store-operated Ca^2+^ channels (20). This activation-induced signal is absent in resting cells. To reveal why the spontaneous Ca^2+^ oscillations are lower in Tregs, we scanned typical calcium regulators using real-time quantitative PCR (qPCR) and found that RyR2, an ER-membrane calcium channel protein, showed markedly reduced mRNA and protein levels in Tregs (Figure 1, D and E, and Supplemental Figure 1B). To see whether the reduction of RyR2 activities resulted in the increased binding to DCs, we performed SCFS with RyR2 knockdown T cells along with several control Ca^2+^ channel knockdowns. RyR2 reduction increased the T cell binding to DCs (Supplemental Figure 1C). To see if RyR2 function is indeed reduced in Tregs, we used its stimulator, 4-CMC, and observed the rate of CMAC (calpain substrate) digestion. While 4-CMC induced calpain activities, Tregs were insensitive to this treatment, confirming the reduced expression of RyR2 (Supplemental Figure 1D). JTV519, a RyR2 inhibitor, reduced Ca^2+^ levels in resting Tconvs to the level of Tregs and elevated the binding force to DCs (Supplemental Figure 1, E and F). In KD assays, *Ryr2*-specific shRNA reduced *Ryr2* mRNA levels (Supplemental Figure 1G). This was correlated with a reduction in the spontaneous Ca^2+^ oscillations in the treated Tconvs (Figure 1F), in line with reduced calpain substrate digestion (Supplemental Figure 1, H and I).

To confirm that this increased binding excluded antigen-specific Tconvs to engage the same DCs, we performed triple-cell binding force analysis (2). Similar to Treg-mediated blockage, as we reported previously (2), *Ryr2*-KD Tconvs inhibited the binding strength between OT-II T cells and OVA-loaded DCs (Figure 1G). Therefore, we confirmed at the single-cell level that a Tconv with reduced RyR2 activity was functionally equivalent to a Treg, in its ability to block Tconv-DC interaction. shRNA-treated Tconvs also suppressed OT-II T cell division in response to OVA-pulsed DCs (Figure 1H), confirming the unique involvement of RyR2.

### Ryr2 is transcriptionally silenced by Foxp3.

**Figure 2:**
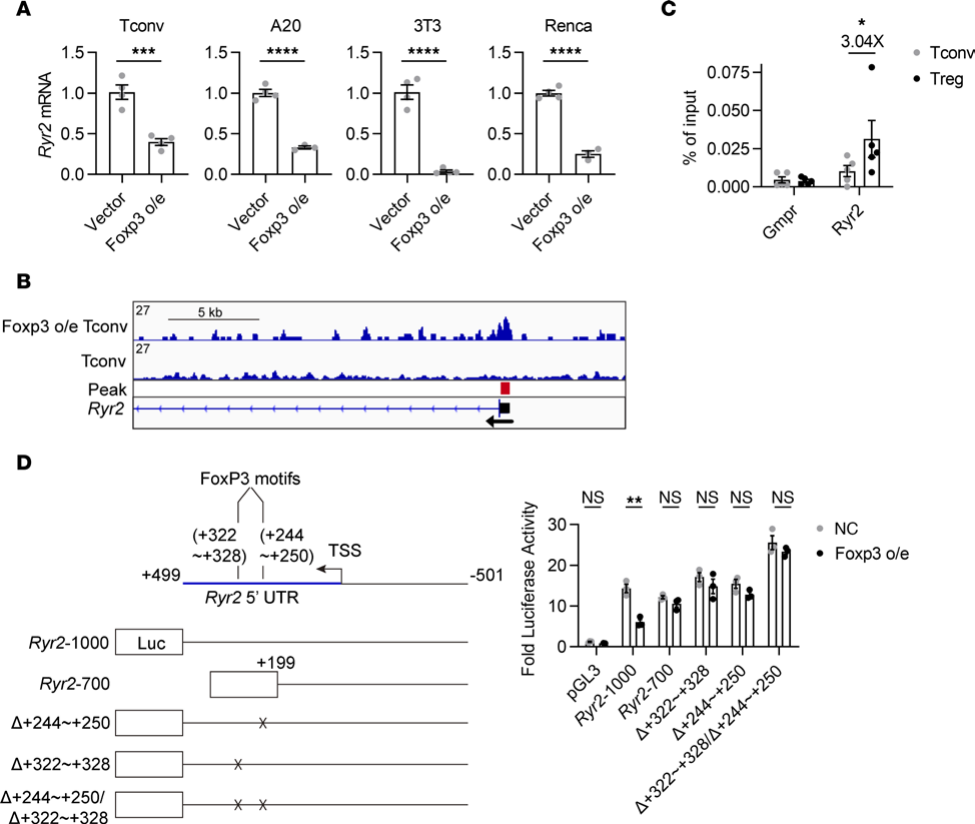
*Ryr2* is transcriptionally silenced by *Foxp3*. (**A**) *Ryr2* mRNA levels in *Foxp3*-overexpressed Tconvs as well as A20, 3T3, and Renca cells were detected by qPCR. *n* = 3–4 per group. *N* = 3. (**B**) Anti-flag-FOXP3 ChIP-seq data set reanalysis was performed in Tconvs with (top track) or without (bottom track) transduction of flag-FOXP3. Shown is the region around the transcription start site (TSS) of the *Ryr2* gene. The red box denotes the position of significant difference identified by the program, which mostly overlaps the promoter region (dark box) of this gene. (**C**) ChIP-qPCR analysis was performed in Tconvs and Tregs cultured overnight in presence of recombinant IL-2 to examine FOXP3-enriched binding in the *Ryr2* promotor region. *Gmpr* was used as negative control. *N* = 3. (**D**) Left: Schematic diagram of the *Ryr2* promoter-luciferase reporter constructs, with deleted positions indicted by ×. Right: Analysis of different truncated or FOXP3-binding sequence consensus-deleted *Ryr2* promoter-driven transcription responses in *Foxp3*-overexpressed 3T3 cells. *n* = 4 per group. *N* = 4. Two-tailed unpaired Student’s *t* test. *P < 0.05; **P < 0.01; ***P < 0.001; ****P < 0.0001.

To confirm the exclusivity of RyR2 reduction in Tregs, it is essential to establish its link to Foxp3. We therefore overexpressed Foxp3 in T, A20, 3T3, and Renca cells. In all cases, the presence of Foxp3 reduced the level of *Ryr2* mRNA, suggesting that Foxp3 targeting of *Ryr2* is autonomous (Figure 2A). Coincidentally, a previous report analyzing the binding of overexpressed Foxp3 in Tconvs produced a whole genome ChIP-Seq data set for gene expression analysis (21). When we used the IGV program to focus this data set on a 40 kb region surrounding the *Ryr2* gene, a signal in the 1.5 kb promoter region was detected over the untransfected control (Figure 2B) (22). To confirm the binding occurs in the native state, we performed ChIP-qPCR analysis with WT Tregs (Figure 2C). Although the signal ratio was not as strong as when Foxp3 was overexpressed (Supplemental Figure 2A), Foxp3 showed a detectable binding to this region. Furthermore, we cloned this region and constructed a luciferase reporter to search Foxp3 binding sites. Truncation experiments narrowed the suppression activity to a defined region between approximately 300 and 500 bp after the TSS site (~200 bp before ATG). We then analyzed this refined segment for any potential Foxp3 binding sites. A GCAGGGG sequence, reported in a previous paper to be targeted by Foxp3 (23), appears twice in the vicinity. When these two sites were depleted, the Foxp3 overexpression lost its ability to suppress luciferase, indicating site-specific Foxp3 binding (Figure 2D). These data indicate that *Ryr2* is indeed under direct control of Foxp3.

### RyR2 deficiency minimally affects T cell development.

**Figure 3:**
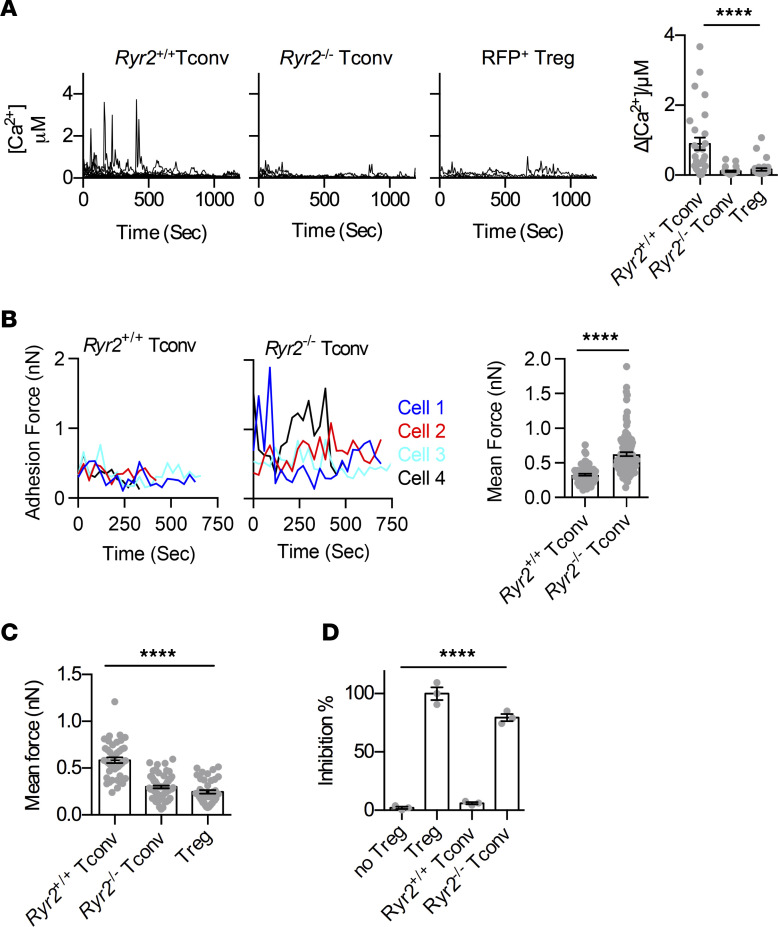
RyR2 deficiency genetically makes Tconvs sticky and suppressive. (**A**) Resting *Ryr2*^+/+^ Tconvs, *Ryr2*^–/–^ Tconvs, and Tregs were loaded with Cal Red R525/650-AM, and Ca^2+^ concentration fluctuations were analyzed. Each line represents one 1 (left). Corresponding amplitude was shown on the right. *n* = 20 per group, *N* = 5. One-way ANOVA with nonparametric Kruskal-Wallis test. (**B**) SCFS force readings for *Ryr2*^+/+^ Tconvs and *Ryr2*^–/–^ Tconvs adhering to DC2.4 and their mean forces. *N* = 3. Two-tailed unpaired Student’s *t* test. (**C**) Mean adhesion forces between OT-II T cells and OVA-pulsed DC2.4 cells that were free or engaged by Tregs, *Ryr2*^+/+^ Tconvs, or *Ryr2*^–/–^ Tconvs on the opposite side of the DC cell bodies. *N* = 3. One-way ANOVA with nonparametric Kruskal-Wallis test. (**D**) *Ryr2*^+/+^ or *Ryr2*^–/–^ Tconv-mediated suppression of OT-II T cell division was analyzed. The relative inhibition efficiencies of Tregs, *Ryr2*^+/+^ Tconvs, and *Ryr2*^–/–^ Tconvs are shown. The inhibition efficiency of Tregs and no Tregs was 100% and 0%, respectively. *N* = 3. One-way ANOVA with nonparametric Kruskal-Wallis test. ****P < 0.0001.

Likely due to its exceptionally large size and complex regulation, thus far, overexpression of RyR2 has not been feasible in primary cells (24). We attempted to produce two mouse models with elevated RyR2 in Tregs: one with RyR2 under a strong viral promoter and one with a Foxp3 conditional knockin where the two Foxp3 binding sites were mutated. Neither of them showed increased RyR2 in Tregs (data not shown). Our attempt to drive the expression of RyR2 with the VP64/dCas9 system as well as several additional methods failed (Supplemental Figure 3A) (25). It should be stressed that we consider this a temporary technical limitation and understand that this might limit our findings; we are continuing to seek new methodologies to stably express RyR2 in sufficient quantities in primary Tregs. However, it was possible to delete *Ryr2* in CD4^+^ T cells with CD4-Cre (Supplemental Figure 3B). *Ryr2* deletion in Tconvs did not result in any overt change in mice, including the rate of development and body weight. Both thymic and peripheral CD4^+^ versus CD8^+^ marker distributions were nearly identical to those of WT mice (Supplemental Figure 3C). The percentage of Foxp3^+^CD4^+^ T cells also remained undisturbed (Supplemental Figure 3D). Other T cell activation indices were also similar, including the frequency of CD44^hi^ T cells, cytokine expression, and CD39/Helios/CD5 levels, as measurement of self-reactivity (Supplemental Figure 3E). The only difference was the percentage of Ki-67^hi^ CD4^+^ Tconvs, a phenomenon likely related to suppressive environment in the *Ryr2*-deficient CD4^+^ T (CKO) cells mice (see below). To further reveal any impact of *Ryr2* deletion in T cell biology, we used 50:50 WT/RyR2 CKO bone marrow to restore the immune system in γ-irradiated recipients. Using CD45.1/CD45.2 markers, we repeated the thymic and peripheral comparisons (Supplemental Figure 3F), and no difference was found. Therefore, the *Ryr2*-specific deletion did not result in developmental defects in T cell subsets. This deletion was accompanied by reduced spontaneous Ca^2+^ oscillations in CD4^+^ T cells (Figure 3A). In comparison to untreated Tconvs, CMAC digestion was reduced in *Ryr2*^–/–^ Tconvs (Supplemental Figure 3G). Similarly, force spectroscopy indicated an increased binding to DCs upon *Ryr2* deletion (Figure 3B). This was accompanied by their enhanced ability to interfere with the DC contact with T cells in the triple-cell force analysis (Figure 3C). More importantly, the *Ryr2*^–/–^ Tconvs behaved similar to Tregs in terms of their ability to suppress OT-II T cell expansion stimulated by antigen^+^ DCs (Figure 3D).

### RyR2 deficiency per se mediates contact-dependent suppression.

**Figure 4:**
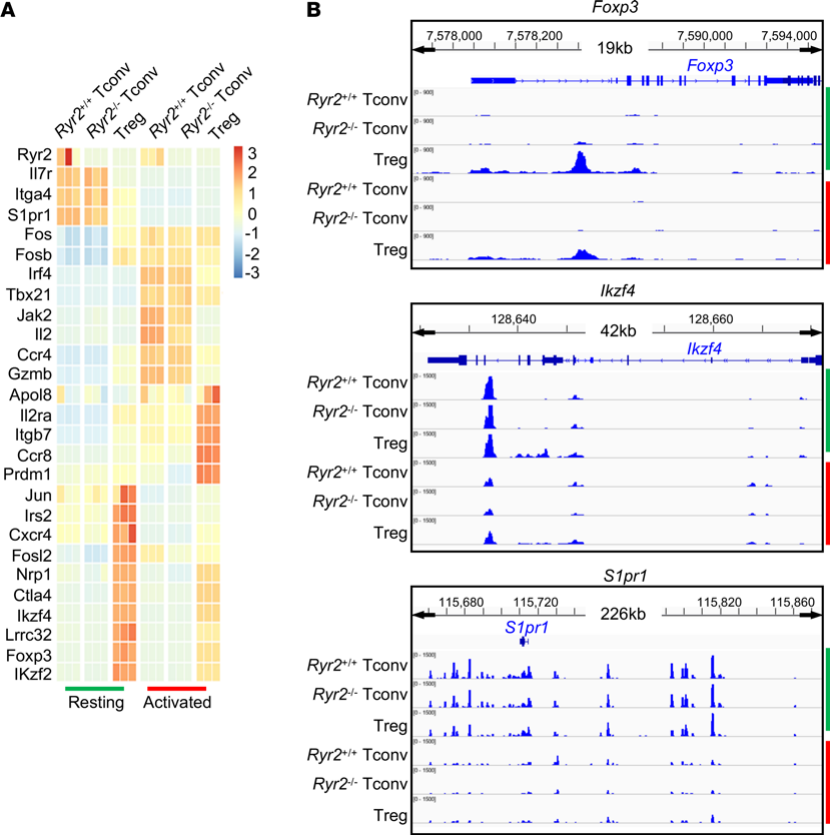
RyR2 deficiency per se mediates contact-dependent suppression. (**A**) Transcriptional differences among *Ryr2*^+/+^ Tconvs, *Ryr2*^–/–^ Tconvs, and Tregs before (green) and after (red) anti-CD3/CD28 activation. Differential expressed genes were ranked by *z* score. Three repeats of each cell were analyzed. (**B**) Chromatin opening status of representative genes in the 3 cells before (green) and after activation (red).

It remained possible that *Ryr2* deletion in Tconvs triggered another suppression mechanism other than our DC occupancy–based hypothesis. We found that *Ryr2*^–/–^ cell surface markers that are potentially associated with Treg functions remained unchanged (Supplemental Figure 4A). TGF-β and IL-10 production was undetectable (Supplemental Figure 4B). To rule out the possibility that *Ryr2* deletion converted Tconvs into Tregs after activation, we stimulated *Ryr2*^–/–^ cells in vitro or in vivo and transferred them into *Rag1*-KO mice; neither scenario showed detectable Treg conversion (Supplemental Figure 4, C and D). To globally analyze the impact of *Ryr2* deletion, we performed RNA-Seq and assay for transposase-accessible chromatin–sequencing (ATAC-Seq) analyses for Tconvs, CKO Tconvs, and Tregs, both under resting state and following activation. RNA-Seq revealed that with the expected reduction of RyR2 in both Tregs and CKO cells, no other suspected suppression function was significantly elevated in CKO cells (Figure 4A). For all of data, we performed Spearman’s correlation and hierarchical clustering among RNA-Seq samples (Supplemental Figure 4E). In summary, among the 3 cell types, the activation versus resting represented the first order (largest) of differences. The second order of differences was between Tregs and Tconvs/CKO cells. The last order was between Tconvs and CKO cells. Therefore, CKO cells and Tconvs were similar, and both of them shared a farther distance to Tregs. ATAC-Seq result showed a similar trend (Figure 4B, Foxp3, Helios and S1P receptor). Again, we failed to identify any major chromatin accessibility change in CKO Tconvs that resembled Tregs. Spearman’s correlation showed the similarity between Tconvs and RyR2 CKO Tconvs, both as a group more distant from Tregs (Supplemental Figure 4F). We also compared the expression level of forkhead family genes and T cell–associated calcium regulators in searching for any compensatory upregulation, and no major change was found in RyR2 CKO cells (Supplemental Figure 4, G and H). Those results seem to suggest that by simply limiting RyR2 expression, the Tconvs gained the ability to suppress DC-mediated T cell activation in vitro, without invoking another overt suppression program.

### RyR2 deficiency–mediated suppression operates in the absence of specific antigen.

**Figure 5:**
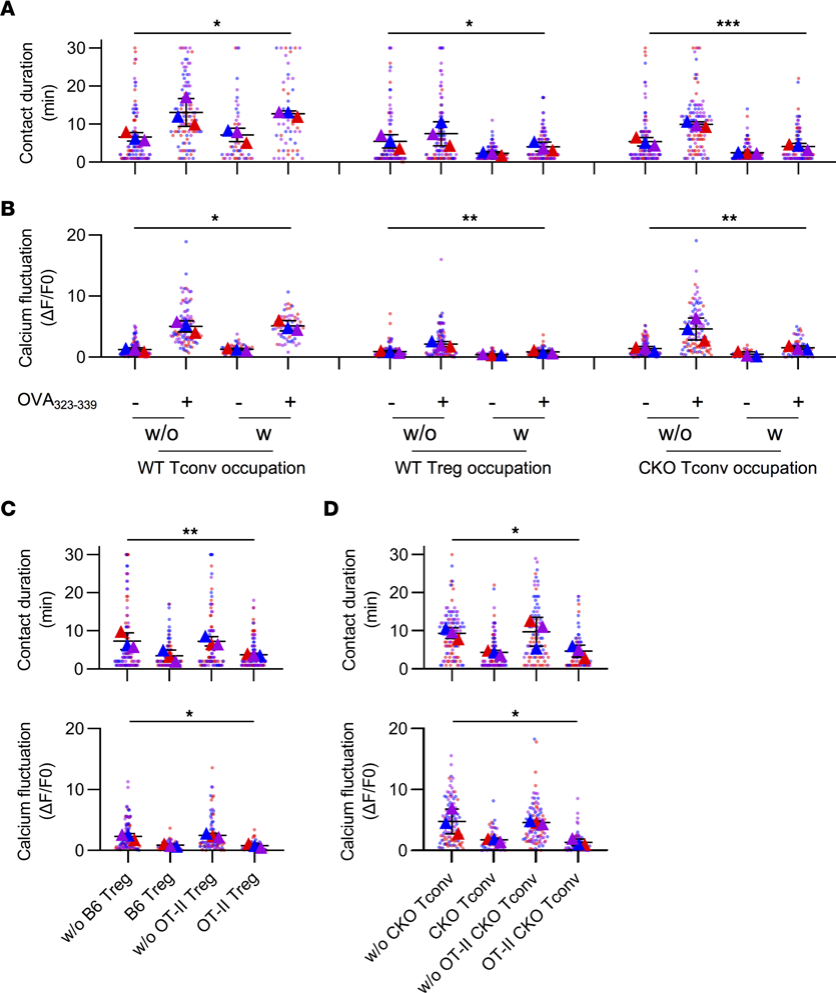
RyR2 deficiency–mediated suppression operates in the absence of specific antigen. (**A**) Contact disruption of responder OT-II cells and DCs by suppressor cells in intravital imaging. WT Tregs and *Ryr2*^–/–^ Tconvs (CKOs) were analyzed as suppressor cells, and WT Tconvs were used as negative control. Both antigen nonspecific and OVA_323–339_-specific contacts were analyzed. 100 contacts were analyzed for each group. w/o, OT-II–DC contacts without suppressor cell occupancy; w, OT-II–DC contacts with suppressor cells on the specific DC. *N* = 3. (**B**) Calcium signal of OT-II T cells during contacts. OT-II cells preloaded with calcium indicator FuraRed were imaged and analyzed for each condition. 100 cells were analyzed for each group. *N* = 3. (**C** and **D**) Contact disruption of OT-II cells and antigen-loaded DCs by suppressor cells. WT Tregs (nonspecific) and OT-II Tregs (specific) were analyzed in **C**. *Ryr2*^–/–^ Tconvs (nonspecific) and OT-II–*Ryr2*^–/–^ Tconvs (specific) were analyzed in **D**. 100 contacts were analyzed for each group. 100 cells were analyzed for each group. *N* = 3. SuperPlots were generated as follows: each dot represents 1 contact or the calcium readout of an individual cell, and each triangle represent 1 batch of an experiment. One-way ANOVA with nonparametric Kruskal-Wallis test. *P < 0.05; **P < 0.01; ***P < 0.001.

A T cell receptor (TCR) repertoire analysis revealed that RyR2 CKO TCR frequencies were almost identical to WT Tconvs and distant from those of Tregs (Supplemental Figure 5A). Therefore, the suppression by RyR2 CKO Tconvs does not appear to involve significant changes in T cell specificity. In our previous reports, we found that Tregs showed prolonged contact with DCs in vivo (2, 7). Another report, mostly using induced, antigen-specific induced Tregs (iTregs), suggested that iTreg suppression intensity was related to antigen specificity (13). We performed the intravital imaging assay on RyR2 CKO Tconvs. As shown in Supplemental Figure 5B, CKO Tconvs exhibited extended contact with DCs in vivo, similar to Tregs. To further reveal if TCR specificity contributed to the extended binding, we also produced ovalbumin 323–339 peptide–specific (OVA_323–339_–specific) RyR2 CKO OT-II T cells and repeated the experiment. Infusion of OVA peptide increased the binding duration between WT OT-II T cells and DCs, yet it had no effect on contact duration between RyR2 CKO OT-II T cells and DCs, suggesting the adhesion to DCs can be inversely regulated by RyR2 and the antigen specificity may not be essential in our settings (Supplemental Figure 5C). To calculate the impact of CKO cell/Treg binding on DC’s ability to interact with antigen-specific responder T cells, we infused OT-II T cells with or without antigen, in the presence of control Tconvs, Tregs, or CKO Tconvs. The antigen increased the contact time between DCs and responder OT-II cells, whether the DC was bound by a control Tconv or not did not change any contact duration for the former pair. However, if the DC was bound by Tregs or CKO cells, the DC/responder OT-II contact time was significantly reduced (Figure 5A). Ca^2+^ reporter activities in the responder OT-II cells translated the contact duration differences into T cell activation intensity (Figure 5B). To address the issue of Treg/CKO cell antigen specificity, we used this 3-cell system again in the constant presence of OVA. In this system, OT-II Tregs and WT Tregs showed identical ability to block DC/responder OT-II cell contact, and the same results were obtained from CKO cells and OT-II CKO cells, suggesting that antigen specificity of thymic Tregs (tTregs) or CKO cells did not affect their suppression efficacy (Figure 5, C and D). Ca^2+^ reporter activities in responder OT-II T cells were consistent with those data (Figure 5, C and D). Therefore, in our system, antigen specificity of Tregs and CKO Tconvs itself does not appear to alter their ability to suppress DC engagement to other T cells. To rule out the possibility that some antigen-specific CKO cells were preferentially expanded over time to mediate the suppression in an antigen-specific manner, we crossed OT-II cells with CKO cells and infused the resulting CD4^+^ T cells into recipient mice. When compared with the WT CKO carrying unaltered TCR, expansion levels were similar, ruling out the possibility of suppression mediated by a selected group of CKO cells (Supplemental Figure 5, D and E). We also considered the possibility that other RyR family members might rise in the absence of RyR2. However, data in Supplemental Figure 4H do not appear to support this notion. As there have been concerns that RyR2 antibodies might have some issues with cross-reactivity to RyR1, we analyzed the expression of the latter and compared it with that in skeletal muscle cells. No RyR1 was detected in T cells regardless of the status of RyR2 (Supplemental Figure 5, F and G).

### RyR2-deficient Tconvs are indistinguishable from Tregs in disease models and scurfy rescue.

**Figure 6:**
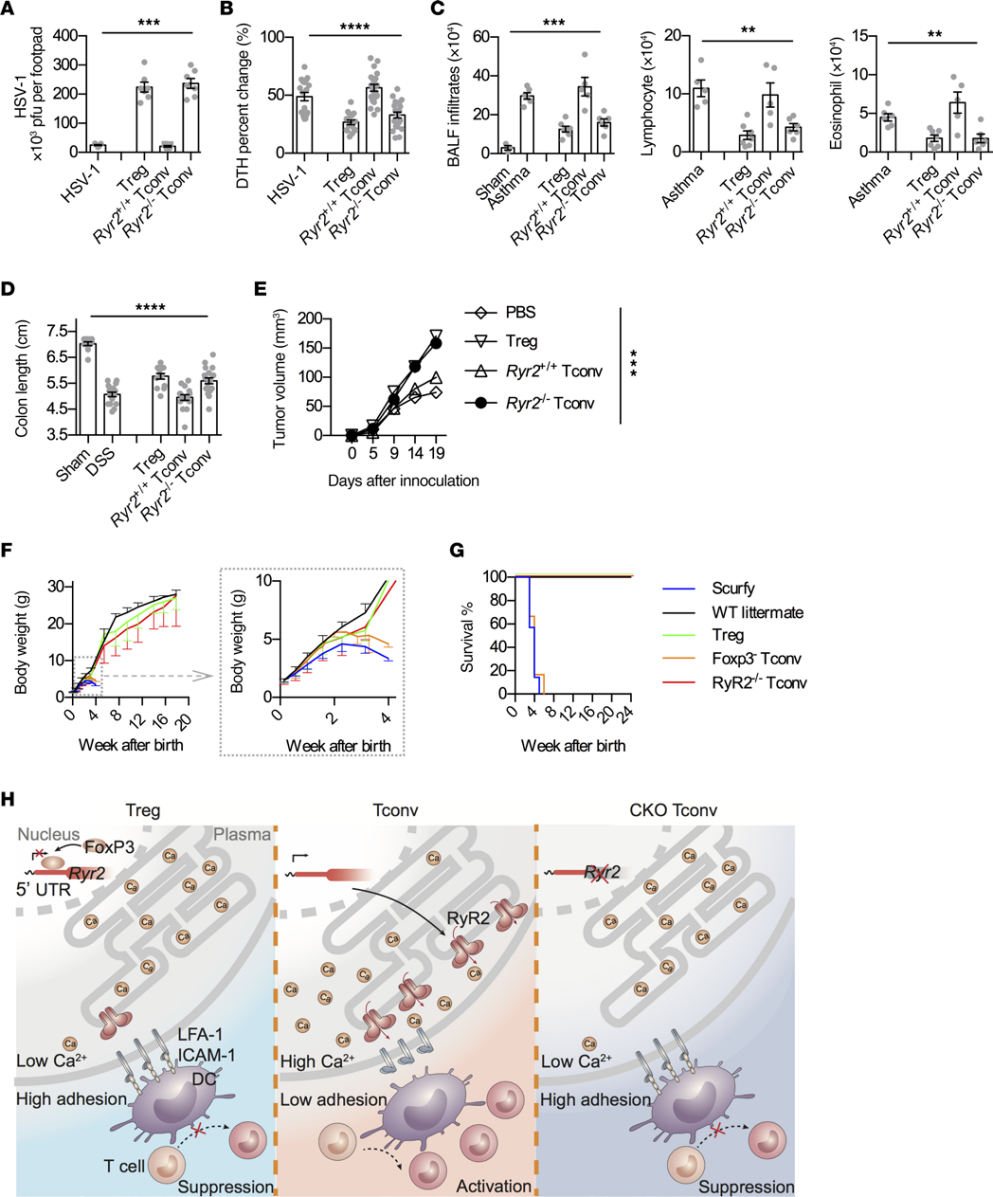
RyR2-deficient Tconvs are indistinguishable from Tregs in disease models and scurfy rescue. (**A** and **B**) HSV-1 footpad infection model. On day 7, HSV-1 titer in footpad tissues (**A**, *N* = 3) and delayed-type hypersensitivity (DTH) response caused by HSV-1 antigen rechallenge in footpad (**B**, pooled data, *n* = 16–21) were analyzed. Footpad thickness without rechallenge was set as 0%. One-way ANOVA with nonparametric Kruskal-Wallis test. (**C**) OVA-induced asthma model. Total infiltrates, infiltrated lymphocytes, and eosinophils in bronchoalveolar lavage fluid (BALF) were analyzed. *N* = 3. One-way ANOVA with nonparametric Kruskal-Wallis test. (**D**) DSS-induced colitis model. Colon length was measured on day 9. Pooled data, *n* = 11–18 mice/group. One-way ANOVA with nonparametric Kruskal-Wallis test. (**E**) MC38 tumor model. Tumor volume was measured. Pooled data, *n* = 10 mice per group. Time point–matched RM 2-way ANOVA. (**F** and **G**) Rescue of scurfy mice. (**F**) Analysis of weight change over time following i.p. transfer of Tregs, *Foxp3*^–^ Tconvs, or CKO Tconvs into newborn scurfy mice. *n* = 10, WT littermate; *n* = 7, scurfy; *n* = 6, Treg; *n* = 6, *Foxp3*^–^ *Ryr2*^+/+^ Tconv; *n* = 6, *Ryr2*^–/–^ Tconvs. Full 20-week rescue and 4-week weight change of scurfy and *Foxp3*^–^ *Ryr2*^+/+^ Tconv are shown. (**G**) Kaplan-Meier survival curves for the mice described in **F**. (**H**) Proposed working mechanism. In Tregs, *Foxp3* expression autonomously suppresses the expression of RyR2, targeting a stretch of sequence roughly 200 bp before *Ryr2* start codon, which results in severely depressed basal calcium oscillation in Tregs. The depressed basal Ca^2+^ level is insufficient to activate m-calpain to cleave LFA-1–anchoring proteins, such as talin, when Tregs contact DCs. This causes Tregs to adhere to DCs with exceedingly strong force, rendering the latter incapable of engaging other Tconvs. **P < 0.01; ***P < 0.001; ****P < 0.0001.

To establish functional equivalency of *Ryr2*^–/–^ Tconvs and Tregs, we tested their behaviors in viral infection, allergic response, autoimmune colitis, and tumor development, with comparison to similarly infused purified Tregs. In a footpad inoculation model (26), HSV-1 pfu counts were increased by about roughly 1 log with either Treg or *Ryr2*^–/–^ Tconv treatment. Control infusion of Tconvs showed no increase over the viral inoculation alone (Figure 6A). With a secondary challenge, this same model has been used to demonstrate delayed-type hypersensitivity response (27). Sensitized footpads were swollen upon second HSV-1 inoculation, with the increase in the thickness was reduced by both Tregs and *Ryr2*^–/–^ Tconvs but not by control Tconvs (Figure 6B and Supplemental Figure 6A). In an OVA sensitization–induced asthma model (28), infusion of Tregs or *Ryr2*^–/–^ Tconvs was equally effective in limiting bronchoalveolar lavage fluid cell number counts, with reductions to a similar degree in both lymphocytes and eosinophils (Figure 6C, sensitization schedule and histology, and Supplemental Figure 6B). In a dextran sodium sulfate–induced (DSS-induced) colitis model, the colon length was reduced. Both Tregs and *Ryr2*^–/–^ Tconvs, but not control Tconvs, were able to reverse the reduction and limit the colon damage (Figure 6D, induction schedule, colon images, and histology, and Supplemental Figure 6C). In MC38 tumor model (29), the infusion of both Tregs and *Ryr2*^–/–^ Tconvs facilitated the tumor growth, with control Tconvs showing no overt effects (Figure 6E). Therefore, in several disease models whereby Tregs are known to show immune regulatory roles, those effects are phenotypically copied by *Ryr2*^–/–^ Tconvs in the absence of Foxp3. The lack of functional Tregs is most evident in Foxp3-deficient (or scurfy) mice, with pervasive inflammation as a result of multiorgan autoimmunity. This pathology leads to death in a defined window of 2–4 weeks of age. If the immune regulatory effects of *Ryr2*^–/–^ Tconvs represent a missing effector function in scurfy mice, *Ryr2*^–/–^ Tconvs should correct those autoimmunities in the systemic absence of Foxp3. scurfy mice (Foxp3^–/–^, C57BL/6) were injected with PBS, Tregs, Foxp3^–^ Tconvs, or *Ryr2*^–/–^ Tconvs 2–3 days after birth. As expected, mice infused with PBS or Foxp3^–^ Tconvs all died within a window of 2–4 weeks after birth (Figure 6, F and G). In contrast, all those infused with Tregs or *Ryr2*^–/–^ Tconvs survived for more than 1 year without any sign of shortened life expectancy. For the histology, control-infused mice were analyzed on week 3, and those from Treg0 and *Ryr2*^–/–^ Tconv-infused groups were analyzed on week 8–12. As expected, PBS- or Foxp3^–^ Tconv-infused mice showed severe thyroiditis, splenitis, pneumonitis, dermatitis, hepatitis, pancreatitis, gastritis, and colitis (Supplemental Figure 6D). The infusion of either Tregs or *Ryr2*^–/–^ Tconvs prevented those pathologies, providing evidence that the regulation of RyR2 is a potentially generalizable effector mechanism of Tregs (Figure 6H). However, it should be recognized that *Ryr2*^–/–^ Tconvs may have some subtle differences from Tregs; for instance, the tissue inflammation scores in the rescued mice were slightly worse in the CKO cell–infused recipients. In the asthma model discussed above, a careful titration with reduced numbers of cells infused showed that Tregs were more efficient than *Ryr2*^–/–^ Tconvs in the disease control. The nature of this nonforce-based inhibition by Tregs is an interesting topic for future investigations.

## Discussion

The observation that Foxp3’s expression in T cells enables its suppressive phenotype supports the idea that Foxp3 is a master regulator of Tregs (30, 31). The complex regulations on Foxp3 are being appreciated at ever-deepening levels. Recent advances on additional regulations, such as via Bcl11b, CDK8/19, and Foxp1, introduce additional complexity (32–34). Even its downstream factors, such as BLIMP1, have been a focal points, as they fine-tune Tregs in adipose tissues by sex-specific factors (35).

In comparison to study of gene regulation of Tregs, studies on Treg effector mechanisms have been more diverse. Trogocytosis of costimulatory molecules on DCs via CTLA4 expressed on Tregs and MHC class II molecule depletion on DCs are two leading hypotheses. Those topological extractions, along with previously reported suppressive cytokines, metabolic blockage, direct T cell cytolysis, and IL-2 deprivation, etc., were all effective in respective settings (1). While those important advancements build our understanding of Tregs, we still cannot come to an unequivocal conclusion about whether there is a specific suppression mechanism. Conceptually, because Foxp3 expression induces a suppressive capacity, the question becomes whether such a switch turns on something fundamentally unique to Tregs. As existing proposed mechanisms are not exclusively controlled by Foxp3, a “truce” can be reached when all these mechanisms are working together to create a network of suppression. However, this synthesis is not ideal, in that the complexity needed to resolve the details of such a Foxp3-originated cooperation is almost experimentally unattainable. Empirically, evidence of those factors working together has been lacking in disease settings or animal models.

We believe that other suppression mechanisms, i.e., biophysical and spatial-temporal mechanisms, may be at work as mechanisms of Treg effector function, particularly considering that the strong binding of Tregs with DCs is an observation that has been repeatedly made by many labs in the field. Our labs proposed in 2017 that spatial occupation of DCs by Tregs limits the former’s ability to engage other T cells (2, 7). Evidence was provided in the form of in vivo contact inhibition and force spectroscopy analysis that the binding essentially blocked the DCs from forming steady contact with Tconvs. We also provided evidence that binding was due to the limited m-calpain activities in Tregs (2). However, why Tregs have limited m-calpain activity was unknown.

In immune cells, Ca^2+^ activation is initiated by IP3, and this is one of the key downstream events of ITAM phosphorylation-mediated enzymatic functions common to FCR, TCR, and BCR. The release of soluble inositol triphosphate by PLC cleavage triggers the opening of IP3R on the ER membrane. This strong Ca^2+^ flux is receptor ligation dependent and unique in immune cells. RyR family members, which have been studied for the control excitation–contraction coupling in muscle cells and synaptic transmission, are ubiquitously expressed and involved in membrane-localized cytoplasmic Ca^2+^ homeostasis (17). Similar to IP3 signals, those events can be trigger dependent (i.e., membrane depolarization). However, RyRs also control basal Ca^2+^ oscillation (36), and those Ca^2+^ activities are gradually coming into focus as being essential to basic cell biology (37). Importantly, Ca^2+^ signaling is likely the most complex regulation in biological systems, with variations in pattern, duration, and subcellular location, and it is not a mere reflection of intensity. A distinct Ca^2+^ signal often reaches its intended target while multiple other Ca^2+^ events are concurrent (38). In T cells, IP3R and store-operated calcium entry (SOCE) channels are the dominant source of Ca^2+^ signaling during activation, with the latter providing the “global” Ca^2+^ waves that can reverberate for minutes to hours. This is in sharp contrast with the “puff-like,” plasma membrane inner leaflet-targeting RyR2 signal that precisely localized at the PM-ER junction (37). As m-calpain is active only in its membrane-associated state, this association may isolate the RyR2–m-calpain regulation from the rest of T cell calcium events.

In this report, we found that Tregs, in comparison to other T cells, lack basal Ca^2+^ oscillation, a phenotype that is a result of severely suppressed RyR2 expression. This baseline Ca^2+^ flux is required for m-calpain activities. Suppression of RyR2 channel activities or genetic blockage of its expression causes Tconvs to behave as Tregs in their suppression. The most critical finding is that RyR2 expression is directly blocked by Foxp3 expression irrespective of cell types, demonstrating a true causative autonomy. *Ryr2* conditional deletion results in the entire pool of Tconvs becoming suppressive, with immune inhibitory capacities indistinguishable from those of Tregs (Figure 6H). Perhaps more tellingly, those Tconvs correct Foxp3 deletion-associated scurfy phenotype and restore immune homeostasis in the absence of Foxp3. Interestingly, in our preliminary analysis, human Tregs also lacked Ca^2+^ oscillation, and deletion of *Ryr2* in human Tconvs resulted in a strong binding to human DCs (our unpublished data), indicating that this mechanism may be of clinical value in the future. In our survey of *Ryr2*^–/–^ Tconvs, factors that may be associated with immune suppression do not appear to be altered, suggesting that this regulation may be independent of previously proposed models. This certainly does not exclude those factors from participating in Treg suppression in other disease models. Yet, the *Ryr2* deletion resulting in an immune suppression similar to that of Tregs suggests that this chain of regulation may be one of the central effector mechanisms of Tregs, fulfilling a missing link in this research field. But caution should also be exercised in how much our findings can be generalized for entire Treg biology. Our model relies on the instantaneous contact between Tregs and DCs, which likely takes away a basal condition for Treg suppression, but many of Treg inhibitory mechanisms are likely late onset and potentially require TCR signaling at some stage of their survival and additional effector functions. Our experimental designs could have unintentionally left out those considerations.

Another point of potential interest is the implication of our work on the debate of whether Tregs must be antigenic specific to exert their immune suppression. For the Treg pool, at least tTregs are believed to originate from the thymus as a group of CD4^+^ T cells carrying highly self-reactive TCRs. Tconv TCRs, on the other hand, are shaped more stringently by the thymic deletion and are therefore unlikely to be the same as those on tTregs (39). As *Ryr2*^–/–^ Tconvs are also potently immune suppressive, similar to Tregs, our results seem to suggest at the moment of suppression, a strong signal via Treg TCR may not be essential, which explains the equal inhibitory potency between antigen-specific and nonspecific Tregs/*Ryr2*^–/–^ Tconvs in our in vivo imaging results. This notion certainly does not rule out that, in certain settings, particularly those in which iTregs rise in the inflammatory milieu, antigen specificity may be quantitatively advantageous, thus providing two layers of suppression to better suit the varying degrees of inflammatory attacks on the host. In addition, our experimental designs are geared toward those responses relying on DC antigen presentation, where antigen-independent occupancies by Tregs may exert a strong inhibition. In the real world, immune stimulation and inflammation are much more complex, and it is possible for Tregs to exert antigen-specific suppression in a disease or time-dependent manner. Therefore, the topic of antigen specificity of Tregs requires much more detailed analysis in the future.

## Methods

### Mice.

All mice were on a CD45.2^+^ C57BL/6J background unless noted otherwise. RyR2^fl/fl^ mice were made at GemPharmatech Co. Ltd. and verified by genotyping. CD11c-DTR/eGFP-transgenic mice were a gift from Yonghui Zhang, School of Pharmaceutical Sciences, Tsinghua University (40). Female Foxp3^+/–^ mice and FOXP3-IRES-RFP mice were obtained in-house. OT-II–transgenic mice, Foxp3^GFP^-transgenic mice, and CD45.1^+^ C57BL/6J mice were obtained in-house (7). CD4-Cre and Foxp3^YFP-cre^-transgenic mice were a gift from Chen Dong, School of Medicine, Tsinghua University (41, 42). WT mice were purchased from Beijing Vital River Laboratory Animal Technology Co. Ltd. RyR2 CKO OT-II mice were generated by crossing RyR2 conditional–KO mice with OT-II–transgenic mice. All mice were bred and housed at Tsinghua University Animal Facilities and maintained under specific pathogen–free conditions and a controlled temperature of 24°C ± 1°C, with a 12-hour light/dark cycle.

### Cell lines and primary cell culture.

DC2.4 cells were a gift from Kenneth Rock of UMass Medical School, Worcester, Massachusetts. Vero cells were from Xu Tan of School of Pharmaceutical Sciences, Tsinghua University. Renca cells were from Guangyu An, Chao-Yang Hospital, Beijing, China. HEK293FT cells were a gift from Wei Guo of School of Medicine, Tsinghua University. MC38 and NIH-3T3 cells were purchased from ATCC. MC38, Renca, NIH-3T3, and Vero cells were cultured in DMEM containing 10% FBS, 100 U/mL penicillin, 100 μg/mL streptomycin. All other cells were grown in RPMI-1640 with the same supplements plus 10 mM HEPES (pH 7.0) and 50 μM β-mercaptoethanol. All cell lines were tested for mycoplasma contamination by PCR analysis. Murine CD4^+^CD25^+^ Tregs and CD4^+^CD25^−^ Tconvs were isolated from spleens using the mouse CD4^+^ T Cell Isolation Kit (STEMCELL Technologies) and mouse CD25 Regulatory T Cell positive selection Kit (STEMCELL Technologies). Tregs and Tconvs sometimes were sorted by FACS from CD4^+^ splenocytes of Foxp3^GFP^ or FOXP3-IERS-RFP–transgenic mice. Murine DCs were isolated from spleens with the mouse CD11c selection Kit II (STEMCELL Technologies). OT-II T cells were isolated from OT-II splenocytes by the mouse CD4^+^ T Cell Isolation Kit and sometimes sorted by FACS with an anti-TCR Vα2 antibody (eBioscience, B20.1).

### Antibody and reagents.

Recombinant human IL-2 was from R&D Systems. The Dual-Luciferase Report Assay System was purchased from Promega. Ultra-LEAF purified anti-mouse CD3ε antibody (145-2C11) and Ultra-LEAF purified anti-mouse CD28 antibody (37.51) for T cell activation were from BioLegend. For flow cytometric analysis, CD62L (MEL-14) monoclonal antibody was purchased from BD Pharmingen, and others (named in the next sentence) were purchased from eBioscience. The following clones were used: CD45.1 (A20), CD45.2 (clone 104), CD3 (17A2), CD4 (GK1.5), CD8a (53-6.7), FoxP3 (3G3), GITR (DTA-1), CD25 (PC61.5), PD-1 (RMP1-30), CTLA-4 (UC10-4B9), CD39 (24DMS1), TIM3 (8B.2C12), LAG-3 (C9B7W), TCR Vα2 (B20.1), CD44 (IM7), CD5 (53-7.3), Nrp1(3DS304M), Helios (22F6), IFN-γ(XMG1.2), IL-4 (11B11), IL-17A (eBio17B7), Ki-67 (SolA15), and Rat IgG2a kappa Isotype Control (eBR2a).

### Western blot.

The cells were collected and lysed with RIPA buffer (Beyotime). The cell lysate was centrifuged and the supernatant was collected. Total proteins were quantified with BCA Protein Assay Kit (Beyotime). After being mixed with 3′SDS loading buffer and boiled for 5 minutes, the proteins were loaded onto 7.5% or 5% PAGE Gels (EpiZyme) or 4%–12% Precast Protein Plus Gels (Yeasen). Then, the proteins were transferred onto a Nitrocellulose membrane (Pall life science/Cytiva) and immunoblotted with indicated primary (Anti-Ryanodine Receptor Antibody, C3-33, Thermo Fisher Scientific, 1:1,000 dilution; anti–β-actin antibody, 2D4H5, Proteintech, 1:20,000 dilution; m-calpain large subunit [M-type] antibody, CST, 1:1,000 dilution) and secondary antibodies (Anti-mouse IgG, HRP-linked Antibody, CST, 1:5,000 dilution). Finally, the immunostained bands were detected by the Super ECL Detection Reagent (Yeasen).

### Real-time qPCR.

Tconvs and Tregs were cultured overnight in presence of recombinant human IL-2 for subsequent experiments unless noted otherwise. Total RNA was extracted from indicated cells using TRIzol reagent (Invitrogen) and first-strand cDNA was synthesized with Reverse Transcriptase M-MLV (TaKaRa). Real-time PCR was performed using Hieff qPCR SYBR Green Master Mix (No Rox) (Yeasen). *gapdh* or 18S RNA was used as reference gene for normalization. The primer sequences were as follows: *gapdh*, 5′-CATCACTGCCACCCAGAAGACTG-3′ and 5′-ATGCCAGTGAGCTTCCCGTTCAG-3′; *18S RNA*, 5′-CGGACAGGATTGACAGATTG-3′ and 5′-CAAATCGCTCCACCAACTAA-3′; *Ryr2,* 5′-ATGGCTTTAAGGCACAGCG-3′ and 5′-CAGAGCCCGAATCATCCAGC-3′; *Ryr1,* 5′-GCACACTGGTCAGGAGTCGTATG-3′ and 5′-GGGTGTAGCACAGGATTTAT-3′; *Ryr3,* 5′-ATCGCTGAACTCCTGGGTTTG-3′ and 5′-TTCATGTCGATGGAACTTAGCC-3′; *Foxp3*, 5′-CCCATCCCCAGGAGTCTTG-3′ and 5′-ACCATGACTAGGGGCACTGTA-3′; *Capn2*, 5′-GGTCGCATGAGAGAGCCATC-3′ and 5′-CCCCGAGTTTTGCTGGAGTA-3′; *Itpr1*, 5′-CGTTTTGAGTTTGAAGGCGTTT-3′ and 5′-CATCTTGCGCCAATTCCCG-3′; *Trpm1*, 5′-ATCCGAGTCTCCTACGACACC-3′ and 5′-CAGTTTGGACTGCATCTCGAA-3′; *Trpm4*, 5′-GGACTGCACACAGGCATTG-3′ and 5′-GTACCTTGCGGGGAATGAGC-3′; *Trpv2*, 5′-TGCTGAGGTGAACAAAGGAAAG-3′and 5′-TCAAACCGATTTGGGTCCTGT-3′; *Cacna2d4*, 5′-GGCAGCAAGTTATCTCCCAG-3′ and 5′-CCACAGGATGATTGGCGTCTT-3′; *Ahnak*, 5′-CAGCGCATCTACACCACGAA-3′ and 5′-CACTTCATGCCTTGGTATCTTGA-3′; *Stim1*, 5′-TGACAGGGACTGTACTGAAGATG-3′ and 5′-TATGCCGAGTCAAGAGAGGAG-3′; *Pkd1*, 5′-CTAGACCTGTCCCACAACCTA -3′ and 5′- GCAAACACGCCTTCTTCTAATGT-3′; *Ncs1*, 5′-AGCAAGTTGAAGCCTGAAGTT-3′ and 5′-GCTGGGGCAGTCCTTAATGAA-3′; *Slc24a3*, 5′-AGCAAGTTGAAGCCTGAAGTT-3′ and 5′-GCTGGGGCAGTCCTTAATGAA-3′; *Hspa2*, 5′-GCGTGGGGGTATTCCAACAT-3′ and 5′-TGAGACGCTCGGTGTCAGT-3′; *P2rx7*, 5′-GACAAACAAAGTCACCCGGAT-3′ and 5′-CGCTCACCAAAGCAAAGCTAAT-3′; *Itgav*, 5′-CCGTGGACTTCTTCGAGCC-3′ and 5′-CTGTTGAATCAAACTCAATGGGC-3′; *Ccdc109b*, 5′-CCACACCCCAGGTTTTATGTATG-3′ and 5′-ATGGCAGAGTGAGGGTTACCA-3′; *Pln*, 5′-AAAGTGCAATACCTCACTCGC-3′ and 5′-GGCATTTCAATAGTGGAGGCTC-3′; *Gsto1*, 5′-ATCCGGCACGAAGTCATCAAC-3′ and 5′-TGACAGATTCGGTGACCAAGT-3′; *Itpr2*, 5′-CTGTTCTTCTTCATCGTCATCATCATCG-3′ and 5′-GAAACCAGTCCAAATTCTTCTCCGTGA-3′; and *Itpr3*, 5′-CTTTATCGTCATCATCATCGTGTTG-3′ and 5′-AGGTTCTTGTTCTTGATCATCTGAGCCA-3′.

### Foxp3 ChIP-qPCR.

ChIP was conducted with the SimpleChIP Plus Sonication Chromatin IP Kit (CST) following the manufacturer’s instructions. Tregs and Tconvs (approximately 2 × 10^7^ cells per assay) cultured overnight were fixed for 15 minutes at room temperature with 1% formaldehyde and then digested with 45 cycles of sonication (Bioruptor). Foxp3 mAb (eBioscience, clone 150D/E4) or control rabbit IgG antibody (CST, clone DA1E) were added to bind. Binding of *Ryr2* promoter was determined by qPCR. The following primer pairs were used: *Gmpr* promoter, 5′-CAGCTGGAACAGCCTTGGAA-3′ and 5′-AAATGTCAAGGCCCCTGTGA-3′ and *Ryr2* promoter, 5′-TGCAGGGGGACCGACC-3′ and 5′-GTCACTGCTAACCAGGATGTTCTA-3′.

### Calcium imaging.

T cells cultured overnight were stained with 2 μM fluo-4 AM (Thermo Fisher Scientific) in Hank’s solution (Coolaber) at 37°C for 30 minutes. After washing and incubation, the cells were allowed to adhere to a poly-L-lysine–coated (0.1 mg/mL; Sigma-Aldrich) round glass slips mounted in a sandwiched chamber made in-house at room temperature. Excess nonadherent cells were removed by flushing with Hank’s solution after 15 minutes. Then, the measurement chamber was placed on an Olympus IX-73 microscope equipped with a 20× (numerical aperture: 0.8) or 40× (numerical aperture: 1.2) Olympus objective. Fluorescence Ca^2+^ signals were recorded as a time lapse for 20 minutes with an interval of 6 seconds. The emission signals at 468–550 nm excited by 488 nm laser were recorded with a charge-coupled device camera (ORCA-AG, Hamamatsu). Data collection was controlled by NIS-Elements 3.0 software (Nikon). The mean fluorescence intensity changes over time for individual cells were analyzed by ImageJ (NIH) and normalized to resting fluorescence F_0_ (Fluo-4 F/F_0_) after subtracting background. Calcium concentration was analyzed based on following equation for fluo-4 (43),
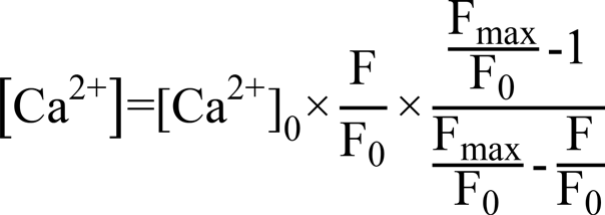


where F_max_ is the fluorescence intensity at 10 mM saturating [Ca^2+^], the cytosolic [Ca^2+^]_0_ is 50 nM for resting T cells (44), respectively.

Ratiometric Ca^2+^ imaging was performed as described previously (24). CD4^+^ T cells were loaded with 5 μM Cal Red R525/650 AM (AAT Bioquest) at room temperature for 30 minutes. The dye-loaded cells were then subjected to wide-field imaging. The Cal Red R525/650 fluorescence dye was excited at 488 nm, and the emitted fluorescence signal was captured at 525 nm (F525) and 650 nm (F650). For Cal Red, the intensity ratios can be converted to intracellular calcium concentration (45), based on the following equation and known dissociation constants (Kd),
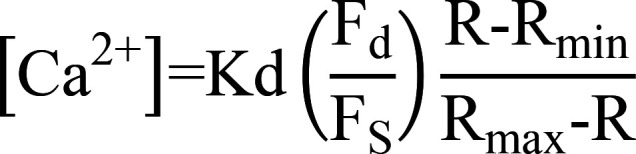


where R represents the fluorescence intensity ratio F525/F650 during the experiment, in which F525 and F650 are the fluorescence detection wavelengths for the ion-bound and ion-free indicator, respectively. R_max_ represents the ion-saturated fluorescence intensity ratio and R_min_ represents the completely ion-free fluorescence intensity ratio. F_d_/F_s_ is the ratio F_max_/F_min_ at the wavelength of the Ca^2+^-free form of the indicator (650 nm).

### Calpain activity measurement.

For measurement of calpain activity, 10^5^ T cells overnight cultured were incubated at 200 μL PBS solution containing 20 μM calpain substrate CMAC (t-BOC-Leu-Met, Thermo Fisher Scientific) at room temperature in dark. The reaction was terminated by 4% PFA (Biosharp) for up to 2 minutes after a 5-minute or 1-minute (for 4-CmC addition) incubation, and then the cells were immediately placed on the ice at least 5 minutes. Calpain activities as ﬂuorescence signals from the digested substrate were determined by Fortessa cytometers (BD Biosciences) via the Hoechst Blue channel. JTV519 (Sigma-Aldrich) was added 30 minutes before the experiment, and 4-CMC was added at the start of assay.

### Flow cytometry.

For surface marker detection, cells were incubated with Fc blocker (CD16/32 antibody; clone 2.4G2) for 5 minutes and then incubated with surface antibody for 15 minutes at room temperature while avoiding light. Stained cells were analyzed directly or, occasionally, after being fixed with 1% paraformaldehyde (PFA) using Fortessa cytometers (BD Biosciences) with FACS Diva software. The Foxp3/Transcription Factor Staining Buffer Set (Invitrogen) was used for intracellular staining of mouse Foxp3, Ki67, and Helios. The Intracellular Fixation/Permeabilization Buffer Set (Invitrogen) was utilized for mouse cytokine detection. Data were analyzed with Flowjo V10.

### ELISA.

For IL-10 and TGF-β detection, 10^6^ purified Tregs, *Ryr2*^+/+^ Tconvs, and *Ryr2*^–/–^ Tconvs were stimulated by anti-mouse CD3ε plus anti-mouse CD28 antibodies. After 72 hours, the supernatants were collected. High-binding 96-well ELISA plates (Nunc) were coated with anti-mouse IL-10 (eBioscience) and anti-human/mouse TGF-β1 capture antibody (eBioscience) at 4°C overnight, respectively. After drying, plates were blocked with 2% BSA in PBS for 1 hour at room temperature. After washing, 100 μL of diluted cell supernatant was added in triplicate wells, followed by incubation for 1 hour at room temperature. Plates were then washed with PBST (0.05% Tween20, Sigma-Aldrich, in PBS) and were incubated with anti-mouse IL-10 and anti-human/mouse TGF-β detection antibody for 0.5 hours at room temperature. TMB (eBioscience) was added (100 mL/well), and the plates were incubated for 10 minutes at room temperature in the dark, followed by the addition of H_2_SO4 (50 μL, 1 M) per well to terminate the reaction. Optical density was immediately read at 450 nm using an ELISA plate reader (Bio-Rad).

### Gene KD and overexpression.

Lentivirus-based shRNA was used to KD specific genes. pLKO.1 vectors purchased from shRNA library platform of Center of Biomedical Analysis of Tsinghua University were used for our all gene KD experiments. All predesigned shRNA sequences were synthesized by Ruibiotech. Then, they were inserted into pLKO.1 vector. Plasmids, including shRNA and the package constructs (pMD2.G and psPAX2), were purified from transformed *E*. *coli* with the EndoFree Plasmid Midi Kit (CWBIO). Lentivirus production was performed according to the manufacturer’s instruction. In brief, 293FT cells were cultured in a 10 cm dish with 60%–80% conﬂuency. Culture media were replaced 2 hours before DNA transfer. 5 μg pLKO.1 vector (with inserted shRNA) was transfected into 293FT cells with 2.5 μg package vectors pMD2.G and 2.5 μg psPAX2 using Neofect DNA transfection reagent (Neofect). 72 hours later, lentiviruses were harvested and added to infect T cells with Polybrene (final concentration 4 μg/mL). 48 hours after the virus infection, cells were sorted with a BD FACSAria cytometer. KD efficiency was verified by real-time PCR. shRNA sequences are as follows: control shRNA, 5′-CCGGcaacaagatgaagagcaccaaCTCGAGttggtgctcttcatcttgttgTTTTTG-3′ and 5′-AATTCAAAAAcaacaagatgaagagcaccaaCTCGAGttggtgctcttcatcttgttg-3′; *Ryr1* shRNA, 5′-CCGGcgtcgcatagaacggatctatCTCGAGatagatccgttctatgcgacgTTTTTG-3′and 5′-AATTCAAAAAcgtcgcatagaacggatctatCTCGAGatagatccgttctatgcgacg-3′; *Ryr2* shRNA, 5′-CCGGccgctaatgaagccatataaaCTCGAGtttatatggcttcattagcggTTTTTG-3′ and 5′-AATTCAAAAAccgctaatgaagccatataaaCTCGAGtttatatggcttcattagcgg-3′; *Ryr3* shRNA, 5′-CCGGccgacatggttcagagagaaaCTCGAGtttctctctgaaccatgtcggTTTTTG-3′ and 5′-AATTCAAAAAccgacatggttcagagagaaaCTCGAGtttctctctgaaccatgtcgg-3′; *Ahnak* shRNA, 5′-CCGGtgccaccatctactttgacaaCTCGAGttgtcaaagtagatggtggcaTTTTTG -3′ and 5′-AATTCAAAAAtgccaccatctactttgacaaCTCGAGttgtcaaagtagatggtggca-3′; *Itpr1* shRNA, 5′-CCGGgcagtaggtaagaagttattaCTCGAGtaataacttcttacctactgcTTTTTG-3′ and 5′-AATTCAAAAAgcagtaggtaagaagttattaCTCGAGtaataacttcttacctactgc-3′; *Stim1* shRNA, 5′-CCGGgcagtactacaacatcaagaaCTCGAGttcttgatgttgtagtactgctTTTTTG-3′ and 5′-AATTCAAAAAgcagtactacaacatcaagaaCTCGAGttcttgatgttgtagtactgct-3′; *Trpm1* shRNA, 5′-CCGGcggagtgaacatgcagcatttCTCGAGaaatgctgcatgttcactccgTTTTTG-3′ and 5′-AATTCAAAAAcggagtgaacatgcagcatttCTCGAGaaatgctgcatgttcactccg-3′; *Trpm4* shRNA, 5′-CCGGgcacatcttcacggtgaacaaCTCGAGttgttcaccgtgaagatgtggTTTTTG-3′ and 5′-AATTCAAAAAgcacatcttcacggtgaacaaCTCGAGttgttcaccgtgaagatgtgc-3′; *Trpv2* shRNA, 5′-CCGGccaaggaacttgtttctatttCTCGAGaaatagaaacaagttccttggTTTTTG-3′ and 5′-AATTCAAAAAccaaggaacttgtttctatttCTCGAGaaatagaaacaagttccttgg-3′; and *Cacna2d4* shRNA, 5′-CCGGtaggaacgcaatggatattaaCTCGAGttaatatccattgcgttcctaTTTTTG-3′ and 5′-AATTCAAAAAtaggaacgcaatggatattaaCTCGAGttaatatccattgcgttccta-3′. Lower case letter sequences are targeting sequences for the intended genes.

For Foxp3 overexpression, pLVX-IRES-mcherry vectors were a gift from Xiaohua Shen of School of Medicine, Tsinghua University. For vector construction, Foxp3 was amplified by PCR using cDNA from Treg total RNA as template a with the following primers: forward primer 5′-ATCGCTCGAGATGCCCAACCCTAGGCCA-3′ and reverse primer 5′-ATCGGAATTCTCAAGGGCAGGGATTGGA-3′. The amplified fragment was gel purified, digested (XhoI and EcoRI), and cloned into the pLVX-IRES-mcherry plasmid, resulting in pLVX-Foxp3-IRES-mcherry construct. Lentivirus production harboring pLVX-Foxp3-IRES-mcherry– and Foxp3-overexpressed cell line generation was performed according to the above KD protocol. Foxp3 expression was verified by real-time qPCR.

To overexpress RyR2, we attempted to use a CRISPR transcriptional activation (CRISPRa) system. All associated vectors, including pLenti-EF1a-dCas9-VP64-blast, pLenti-sgRNA(MS2)-EF1a-zeo, and pLenti-MS2-P65-HSF1-2A-Hygro, were a gift from Qiaoran Xi, School of Life Sciences, Tsinghua University. BleoR sequence in pLenti-sgRNA(MS2)-EF1a-zeo plasmid was replaced with EGFP to generate Lenti-sgRNA (MS2)-EF1a-EGFP backbone plasmids. For designing and cloning sgRNA, in brief, 20-nucleotide gRNA sequences targeting the promoter of *Ryr2* were designed with the CRISPR design prediction tool (ref. 46; http://crispor.tefor.net/). Selected gRNAs were cloned into Lenti-sgRNA (MS2)-EF1a-EGFP backbone plasmid by following the SAM target sgRNA cloning protocol. We generated sgRNA insertion by annealing each oligo: sgRNA-2, 5′-CACCGCCTCCGGGCCGCCAAACCCG-3′ and 5′-AAACCGGGTTTGGCGGCCCGGAGGC-3’; sgRNA-4, 5′-CACCGGTGCCCTTCCTGACCTCAAG-3′ and 5′-AAACCTTGAGGTCAGGAAGGGCACC-3′; and sgRNA-5, 5′-CACCGAGGAGCTCAGCTTCCCGCTG-3′ and 5′-AAACCAGCGGGAAGCTGAGCTCCTC-3′.

The annealing reaction was performed using T4 polynucleotide kinase (NEB) under the following conditions: 37°C for 30 minutes; 95°C for and 5 minutes; a ramp up to 25°C at 5°C/min. Diluted annealing product (1:10) was mixed with backbone vector at 1:2.5 mass ratio in Golden Gate reaction using BsmBI restriction endonuclease (NEB) and T4 DNA ligase (NEB). The program was performed on a thermal cycler: 37°C for 5 minutes, 20°C for 5 minutes and repeated for 30 cycles; and 60°C for 5 minutes. Through the above steps, a gRNA fragment was inserted between the U6 promoter and sgRNA-MS2 scaffold to result in the pLenti-sgRyR2 (MS2)-EF1a-EGFP construct. Then, pLenti-EF1a-dCas9-VP64-blast, pLenti-sgRyR2(MS2)-EF1a-EGFP, and pLenti-MS2-P65-HSF1-2A-Hygro were cotransfected into MC38 cells, and RyR2 expression was verified by real-time qPCR.

### Atomic force microscopy–based SCFS.

The experiments were performed as previously described using a JPK CellHesion unit (2, 47). In brief, to measure T cell–DC adhesion forces in bicellular system, DC2.4 cells were cultured on untreated glass disks. T cells were treated with 200 U/mL recombinant human IL-2 overnight. The disks were moved into an atomic force microscopy–compatible (AFM-compatible) chamber and mounted on to the machine stage. A clean cantilever was coated with CellTak (BD) and then used to glue individual T cells added to the disk. The AFM cantilever carrying a single T cell was lowered to allow T cell contact with an individual DC and interaction for 15 seconds before being moved upward, until 2 cells were separated completely. The force curves were acquired. The process was then repeated. For the triple-cell system, DC2.4 cells cultured on glass disks were pulsed with 100 μg/mL soluble OVA protein for 4 hours before experiments. IL-2–treated Tregs/Tconvs were treated with IL-2 overnight and were stained with 10 μM CFSE, and DC2.4 cells were incubated with these ﬂuorescence labeled Tregs or Tconvs for about 20 minutes before unlabeled OT-II T cells were added. Treg/Tconv-DC couples identified with an UV flashlight were then approached by the cantilever tip carrying an OT-II T cell. Treg/Tconv-mediated suppression of OT-II–DC adhesion was assayed. In each cycle, the AFM cantilever carrying a single T cell was lowered by 0.5–2 μm increments until the first force curve was generated. The T cell on the cantilever was then allowed to interact with the DC for 15 seconds before being moved upward, until 2 cells were separated completely. The incubator chamber in which the machine was housed was conditioned at 37°C and at 5% CO_2_. In all experiments, a minimum of 14 force curves were collected for further analysis. The force curves were processed using the JPK image processing software. Only round and robust cells were selected for AFM gluing. For each SCFS experiment, a T cell–DC pair was used to generate force readings from each up and down cycle over a period of several minutes; these readings are plotted. At least 3 such pairs were used for each condition.

### Suppressive function in vitro.

10^4^ purified DCs from splenocytes were pulsed with 2 μg/mL OVA_323–339_ peptide, and then suppressor cells (2 × 10^4^ Tregs or *Ryr2*^+/+^ Tconvs or *Ryr2*^–/–^ Tconvs or *Ryr2* KD Tconvs) were added onto DCs to occupy the latter for 30 minutes. CD25^-^ OT-II Tconvs were stained by CellTrace CFSE (Thermo Fisher Scientific). 2 × 10^4^ OT-II Tconvs were mixed in DC-suppressor cell culture to compete with OVA-loaded DCs occupied by suppressor cells in a 96-well U-bottom plate. The proliferation of OT-II T cells was assessed by CFSE dilution by Fortessa flow cytometry (BD Biosciences). Inhibition percentage was calculated using 1 – proliferation% and then normalized no-Treg group as 0% inhibition and the Treg group as 100% inhibition.

### RNA-Seq and ATAC-Seq.

For transcriptome comparison, total RNA of Tconvs and Tregs cultured overnight from WT or CKO mice were extracted and sequenced (Annoroad). Anti-CD3/CD28–activated cells were sequenced as well. For chromatin opening comparison, Tn5-based libraries were built from fixed cells and sequenced (TruePrep DNA Library Kit TD501, Vazyme). RNA-Seq FPKM files were correlated with the Pearson correlation method, and then hierarchy clustering was calculated and heatmaps were plotted using Euclidean distance. ATAC-Seq data were analyzed with deeptools (version 2.0; https://deeptools.readthedocs.io/en/develop/#) and then correlated and clustered by Spearman’s correlation. DEGs were visualized with log_10_ FPKM.

### Visualization and analysis of ChIP-seq data.

Visible ChIP-seq data were downloaded from GEO data sets (Foxp3 in Tconvs, GSM989036; Foxp3 in Tconvs transduced to express flag-Foxp3, GSM989034). Visualization of ChIP-seq data was facilitated by IGV (v2.4.14; https://igv.org/doc/desktop/) with mouse reference genome mm8. Gene tracks were generated as screenshots in specific gene loci.

### Dual-luciferase report assay.

Dual-luciferase report assay was established as described previously (48). Murine *Ryr2* reporter plasmid was constructed by subcloning 1,500 bp of the *Ryr2* promoter into a luciferase expression pGL3 vector. The whole promoter sequence and truncated ones were synthesized, and the plasmids were confirmed by sequencing. 1.25 × 10^5^ Foxp3-overexpressed 3T3, Renca, or A20 cells were cotransfected with 300 ng *Ryr2* reporter and Renilla luciferase reporter plasmids using Neofect (Neofect). 36 hours after transfection, cell lysates were prepared and analyzed using the Dual-Luciferase Report Assay System (Promega).

### Intravital imaging.

For basal contact dynamics between T cells and DCs in vivo, *Ryr2*^+/+^ Tconvs (Celltrace Far Red labeled), *Ryr2*^–/–^ Tconvs (TAMRA labeled), and WT Tregs (Far Red-TAMRA duo labeled) cells were i.v. transferred into CD11c-DTR/eGFP-transgenic mice, at 1:1:1 ratio, 3 × 10^6^ cells each. Approximately 12–18 hours after transfer, inguinal lymph nodes were exposed and imaged at 37°C in the intrafollicular zone. 4D (3D stack + time) videos were imported into Bitplane Imaris software and analyzed. DC channel (eGFP-positive) area was calculated as surfaces and then set as the reference plane for T cell migration. Contacts were designated as T cell–DC distance <1 μm, and contact duration was designated as continuous contact until release (T cell–DC distance, >1 μm). For antigen-specific interactions, 6 hours after Far Red OT-II Tconv transfer, 50 μg OVA_323–339_ + 1 μg LPS was injected subcutaneously in right abdomen. Both draining inguinal lymph node (right) and control node (left) were imaged at approximately 12–18 hours. For contact disruption of OT-II cells and DCs by suppressor cell occupation (Ryr2^+/+^ Tconvs, *Ryr2*^–/–^ Tconvs, WT Tregs, OT-II Tregs and *Ryr2*^–/–^ OT-II Tconvs), suppressor cells were labeled with 5 μM CellTrace Yellow (Thermo Fisher Scientific), and OT-II responder cells were labeled with 5 μM CellTrace FarRed (Thermo Fisher Scientific). A 1:1 mixture of both cells (5 × 10^6^ each) was i.v. transferred into CD11c-DTR/eGFP-transgenic mice. For calcium fluctuation experiments, OT-II responder cells were first labeled with FarRed and then loaded with 5 μM calcium indicator FuraRed and transferred. Videos were analyzed with Imaris (Bitplane), and contacts between DC and responder T cells with or without suppressor cell occupation were analyzed.

### Mixed bone marrow chimera.

CD45.1 mice were bred with CD45.2 C57BL/6 mice to generate CD45.1/2 mice (the first-generation animals) expressing both the allelic variants. Mixed bone marrow chimeras were generated by i.v. injecting 5 × 10^5^ CD45.2 CKO bone marrow cells in conjunction with an equal number of CD45.1 control bone marrow cells into lethally x-ray irradiated (2 doses of 5.5 Gy, 2 hours apart) 5-week-old CD45.1/2 recipient mice. Mice were sacrificed 8 weeks after bone marrow transplantation. Thymi, spleens, and lymph nodes from mixed bone marrow chimeras were harvested for characterization of lymphocyte development.

### TCR repertoire.

To assess whether *Ryr2* deletion affects T cell thymic selection, T cell receptor Vβ chains of splenic CD4^+^ T cells from CKO and control mice were assessed using a commercial mouse TCR Vβ screening panel (BD Biosciences). Flow cytometry data collection was performed on an LSR II (BD Biosciences). Data on the expression of TCR vβ chains in splenic CD4^+^ T cells for peripheral TCR usage and the frequencies of CD4^+^ Tconvs and Tregs expressing commonly utilized TCR Vβ chains were analyzed using FlowJo V10.

### H&E histology.

Mice were sacrificed by necropsy. Skin, ears, livers, and other tissues were all fixed in 4% neural buffered formalin at 4°C for over 48 hours before processing. Then samples were embedded in paraffin. Five- to six-micrometer thick slides were cut. All slides were stained with H&E. Inflammation was scored as follows: 0, no inflammatory infiltration; 1, sparse infiltration; 2, obvious infiltration; 3, massive infiltration with normal tissue morphology; 4, massive infiltration with tissue deform; and 5, complete tissue destruction.

### HSV-1 infection model.

Herpes infection was induced by 10^6^ pfu HSV-1 (F strain) in 20 μL PBS into hind footpads at day 0. At 3 days after injection, cells (Tregs, *Ryr2*^+/+^ Tconvs, or *Ryr2*^–/–^ Tconvs) were adoptively transferred into the footpad with 2 × 10^5^ cells/20 μL PBS. Virus titer was tested 7 days after injection with homogenized footpad tissue on Vero cells. For delayed-type hypersensitivity, the right footpad was rechallenged with UV-inactivated HSV-1 (10^6^ pfu/20 μL PBS) at 6 days after injection, and then footpad swelling was measured at 7 days after injection, with the left footpad as control.

### Asthma model.

Airway inflammation was induced by OVA. At day 0 and 14, 1 i.p. injections of OVA plus alum adjuvant (100 μg + 4 mg in 200 μL PBS) were given to sensitize mice. Intratracheal OVA rechallenge (50 μg in 50 μL PBS) was repeatedly given at back of the tongue on days 21, 23m and 25. At day 23, 10^6^ cells (Tregs, *Ryr2*^+/+^ Tconvs, or *Ryr2*^–/–^ Tconvs) were adoptively transferred i.v. in 100 μL PBS. Bronchoalveolar lavage fluid (BALF) infiltrates and histology were analyzed at day 32.

### DSS-induced colitis model.

DSS-induced murine experimental colitis was established as described previously (49, 50). Briefly, 3 × 10^6^ *Ryr2*^–/–^ Tconvs, WT Tconvs, or Tregs were transferred into 6-week-old male C57BL/6 mice i.v. The second day, colitis was induced by oral administration of 4% DSS (w/v) (Yeasen, MW = 36,000–50,000 Da) in drinking water for 7 days followed by normal drinking water. Normal control mice were treated with PBS and were given normal drinking water. The mice were sacrificed on day 10, colons were dissected, and colon length was measured. The colons were fixed at 4°C over 48 hours in 4% PFA for subsequent H&E staining. The colon sections were examined by 3DHISTECH Pannoramic SCAN.

### MC38 tumor model.

The grafted tumor mouse model was established as previously described (51). Briefly, MC38 colon cancer cells were washed twice in PBS, and 5 × 10^5^ MC38 cells in 200 μL PBS were coinjected with 10^6^ T cells s.c. into the abdomen of 6-week-old male C57BL/6 mice after barbering. Tumor growth was monitored using a slide caliper approximately every 4–5 days from day 7 after tumor injection. The volume was calculated as (length × width × width)/2. Group 1 consisted of MC38 + PBS; group 2 consisted of MC38 + Tconvs; group 3 consisted of MC38 + Tregs; and group 4consisted of MC38 + *Ryr2*^–/–^ Tconvs. There were 10 mice in each group.

### Rescue of scurfy mice.

The rescue of Foxp3-deficient mice was established as previously described (52, 53). Scurfy mouse genotypes were analyzed at birth with pamprodactylous-clipped tissue routinely. First, adoptive transfer of 5 × 10^6^ purified cells (Tregs, Foxp3-deficient scurfy Tconvs, or CD25^-^*Ryr2*^–/–^ Tconvs) was performed on day 2 or 3 of life in 50 μL PBS for i.p. injection into newborn syngeneic scurfy mice. Then, in the first 2 weeks, cells transfer was performed every 3 or 4 days and then once every 2 weeks. When transferring, the body weight of scurfy and male WT littermates was recorded and survival was monitored. Genotyping for the sf mutant gene was conducted by PCR and verified by sequencing. Primers for *Foxp3* PCR were 5′-CATCCCACTGTGACGAGATG-3′ and 5′-ACTTGGAGCACAGGGGTCT-3′. For the histology, PBS and Foxp3^–^ Tconv-infused mice were analyzed on week 3, and those from Treg- and *Ryr2*^–/–^ Tconv-infused mice were on week 8–12. Skin, ear, liver, and other tissues were all fixed for H&E staining.

### Statistics.

The numbers of experimental repeats are shown in figure legends. Two-tailed unpaired Student’s *t* test was used for comparing endpoint means between 2 groups. One-way ANOVA with nonparametric Kruskal-Wallis test was used for multiple comparisons among 3 or more groups. SuperPlots of data were generated as previously described (54). Data are presented as the mean ± SEM, unless indicated otherwise. Calculation and graphing were done with Prism (GraphPad Prism 8). *P* values of less than 0.05 were considered significant.

### Study approval.

All animal experiments were conducted in accordance with governmental and institutional guidelines for animal welfare and were approved by the Tsinghua University IACUC.

### Data availability.

Sequencing data can be accessed via GEO (GSE237032). Values for all data points in graphs are reported in the Supporting Data Values file.

## Author contributions

Co–first authorship order was decided by weighing productivity and significance of work; to be specific, Xiaobo Wang carried cellular and molecular studies to make concrete progress, SG carried sequencing and animal studies to show the translational value of this work, and JM performed the initial proof-of-concept experiments for this study. Xiaobo Wang and JM performed all the experiments unless noted otherwise. Xiaobo Wang and SG designed and performed all animal experiments with help from JG, ZZ, and XM. JM and NK performed AFM-related experiments. XL performed ChIP-qPCR and in vitro suppression. XX performed calcium imaging. HL and Ying Xu performed shRNA screening of KD cells. BZ analyzed and visualized ChIP-seq data. Yanni Xu and Xin Wang performed RyR2 protein detection. XS and SG performed intravital imaging assays. DZ and Xiaoting Wang performed cytokine detection. Xiaoting Wang and NN performed transcription analysis of calcium channel genes. RS performed histology analysis. RW assisted with RyR2 function analysis. JG provided scurfy and Treg biology expertise. SRWC provided expert insight in RyR2 regulation. XZ provided critical review of Treg biology. TX provided biophysical mechanisms of cell adhesion. HQ provided overall critical insight. XH provided expert insight on gene regulation of RyR2. YS was responsible for the overall design and wrote the manuscript with input from XH, HQ, and RW.

## Supplementary Material

Supplemental data

Supporting data values

## Version 1 12/15/2023

Electronic publication
