# Supplementary material for: Foxp3-mediated blockage of ryanodine receptor 2 underlies contact-based suppression by regulatory T cells
Source: J Clin Invest. 2023 Dec 15;133(24):e163470. doi: 10.1172/JCI163470 (PMC10721146; doi:10.1172/JCI163470)
Supplement: Supplemental data [file jci-133-163470-s104.pdf]

Supplemental Figure 1

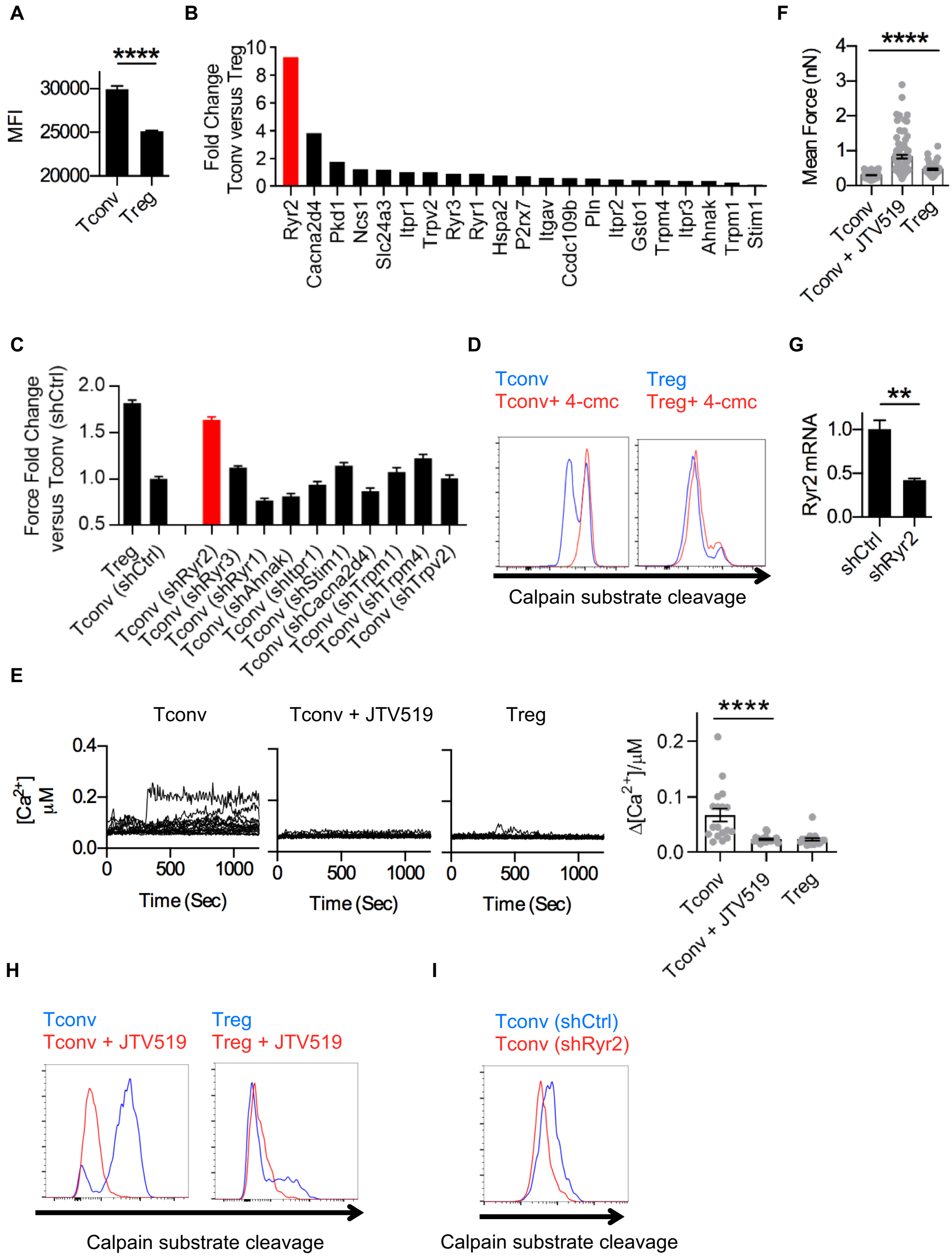

Supplemental Figure 1. Reduced RyR2 activity is the basis of contact-dependent suppression. (**A**) Quantification of the mean fluorescence intensity (MFI) of Ca^{2+} signals of Tconvs and Tregs under resting state with Fluo 4. N=5. 2-tailed unpaired Student's t test. (**B**) qPCR analysis of Ca^{2+} regulation-associated protein levels between Tregs and Tconvs, ranked by the fold of differences. (**C**) Relative fold change of mean forces. IL-2-treated Treg cells and shRNA samples of *Ryr2*, *Ryr1*, *Ryr3*, *Ahnak*, *Itpr1*, *Stim1*, *Cacna2d4*, *Trpm1*, *Trpm4* and *Trpv2* genes were normalized to control Tconv cells (shCtrl). Force were measured with DC2.4 cells. n=4 per group. N=3. 2-tailed unpaired Student's t test. (**D**) Analysis of calpain activities in Tconv (left) and Treg (right) cells treated with 4-CMC as determined by CMAC digestion. N=4. (**E**) Resting Tconv loaded with Fluo-4 AM were treated with 5 μM JTV519 for 30 min then Fluo-4 fluorescence signal over time was recorded. Untreated Tconvs and Tregs were as control. The change of intracellular free Ca^{2+} concentration [Ca^{2+}] over time were shown. Corresponding amplitude was shown in the middle. n=20, N =5. (**F**). Adhesion force of Tconv treated with JTV519 was shown in the right corresponding (**E**). N=3. One-way ANOVA with nonparametric Kruskal-Wallis test. (**G**) Analysis of *Ryr2* gene shRNA knockdown efficiency in Tconv cells isolated from $Foxp3^{GFP}$ mice using qPCR. n=4 per group. N=3. 2-tailed unpaired Student's t test. (**H** and **I**) Analysis of the calpain activities in JTV519-treated Tconvs (**H**), Tregs (**H**) and *Ryr2* knockdown Tconvs (**I**) cells by CMAC digestion. N=4.

Supplemental Figure 2

A

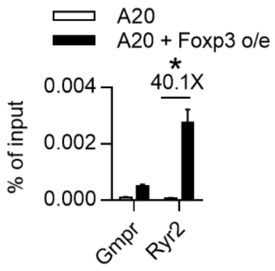

Supplemental Figure 2. *Ryr2* is transcriptionally silenced by Foxp3. (**A**) ChIP-qPCR analysis was performed in *Foxp3*-overexpressed A20 cells to examine FOXP3 enriched binding of *Ryr2* promotor region, with *Gmpr* as negative control. N=3. 2-tailed unpaired Student's t test.

Supplemental Figure 3

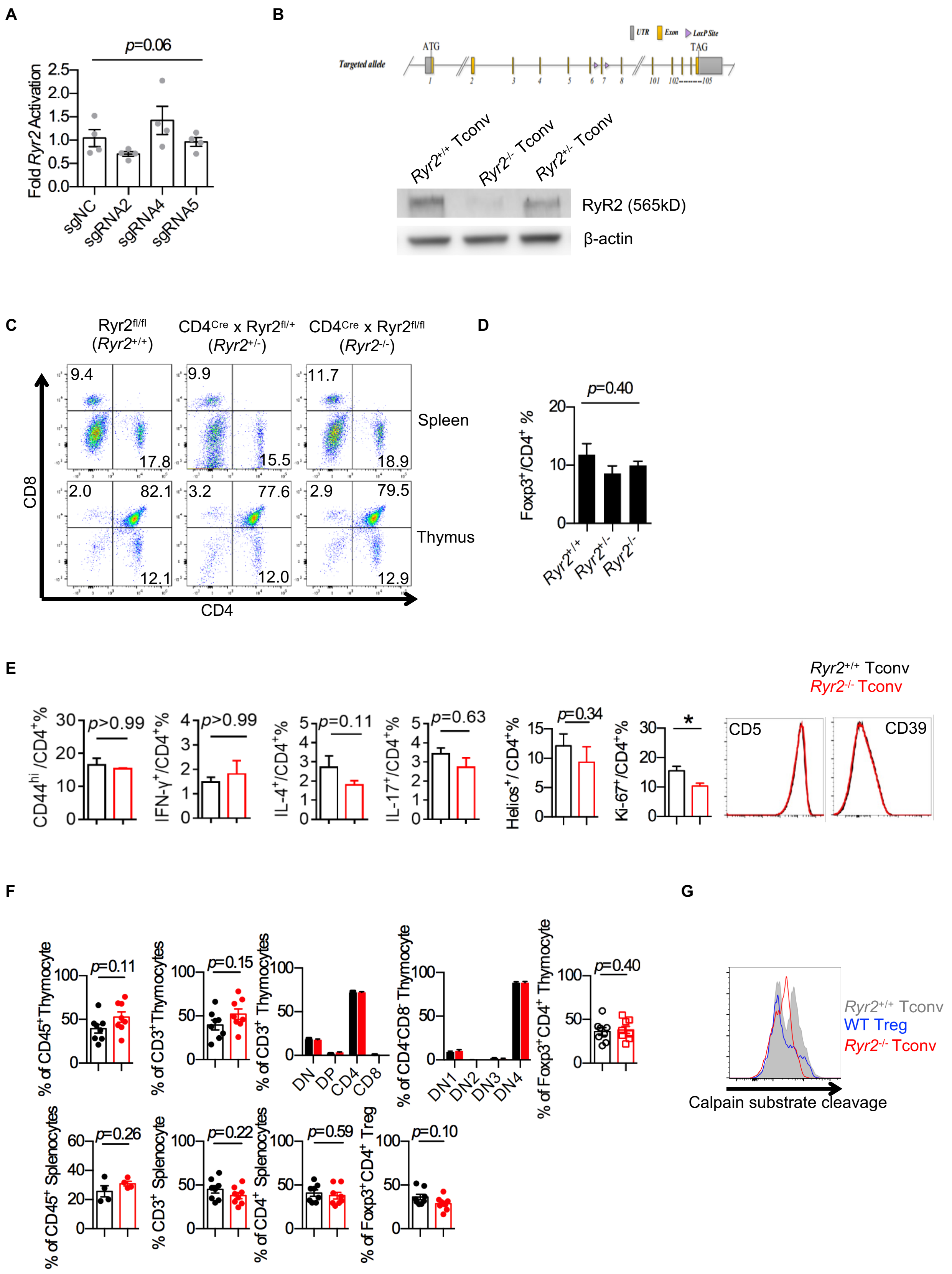

Supplemental Figure 3. RyR2-deficiency minimally affects T cell development. (**A**) Fold activation of *Ryr2* transcripts in MC38 cells by three sgRNAs using an engineered CRISPR-Cas9 complex (CRISPRa system) were shown. qPCR analysis was performed. n=4 per group. N=3. One-way ANOVA with nonparametric Kruskal-Wallis test. (**B**) The construction of conditional knockout mice with intended Cre-dependent deletion of exon 7 in *Ryr2* gene (left) was shown. *Ryr2* of $CD4^+$ T cells was deleted in CKO mice examined by Western Blot (right). (**C**) Distribution of $CD4^+$ and $CD8^+$ T cells in spleen and thymus of *Ryr2*$^{fl/fl}$ (*Ryr2*$^{+/+}$) mice, CD4-Cre/*Ryr2*$^{fl/+}$ (*Ryr2*$^{+/-}$) mice and CD4-Cre/*Ryr2*$^{-/-}$ (*Ryr2*$^{-/-}$) mice was detected by flow cytometry. N=3. (**D**) The proportion of $CD4^+Foxp3^+$ cells in $CD4^+$ splenocytes in mice shown in C. n=3 per group, N=3. One-way ANOVA with nonparametric Kruskal-Wallis test. (**E**) The frequency of $CD44^{hi}$, $IFN\gamma^+$, $IL\text{-}4^+$, $IL\text{-}17^+$, $Helios^+$ and $Ki67^+$ T cells and CD39/CD5 expression levels of $CD4^+$ splenic T cells from CKO vs control mice were measured by FACS, n=3 per group. N=3. 2-tailed unpaired Student's t test. (**F**) Mixed bone marrow chimera. Thymus and spleens from mixed chimeras were harvested for characterization. Shown are *Ryr2*$^{+/+}$ CD45.1 and *Ryr2*$^{-/-}$ CD45.2 percentages in $CD45^+$, $CD3^+$, $CD4^-CD8^-$, $CD4^+CD8^+$, $CD4^+$, $CD8^+$ thymocytes, and $CD25^-CD44^+$ (DN1), $CD25^+CD44^+$ (DN2), $CD25^+CD44^-$ (DN3), $CD25^-CD44^-$ (DN4) $CD4^-CD8^-$ thymocytes, $Foxp3^+$ $CD4^+$ thymocytes (upper), $CD45^+$, $CD3^+$, $CD4^+$, $CD8^+$ and $Foxp3^+$ $CD4^+$ splenocytes (lower). One dot represents one mouse. 2-tailed unpaired Student's t test. (**G**) Calpain activities in Treg, *Ryr2*$^{+/+}$ and *Ryr2*$^{-/-}$ Tconv cells were measured by using CMAC digestion.

Supplemental Figure 4

A

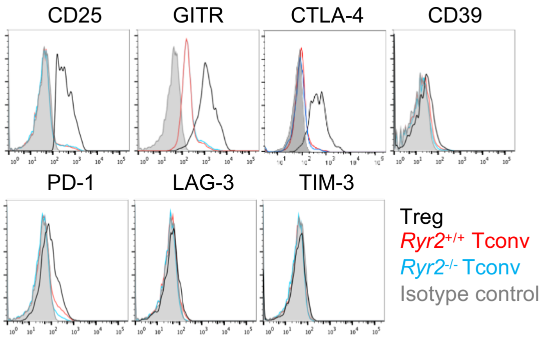

B

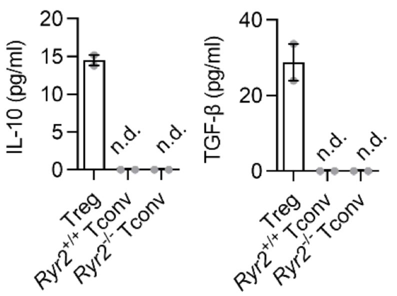

C

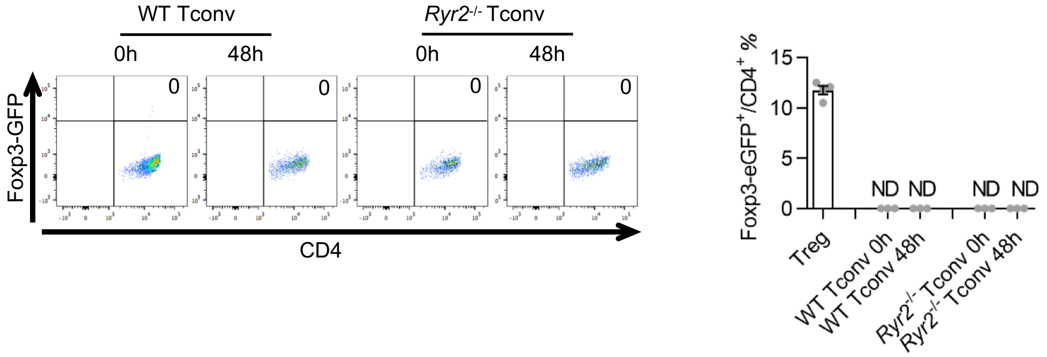

D

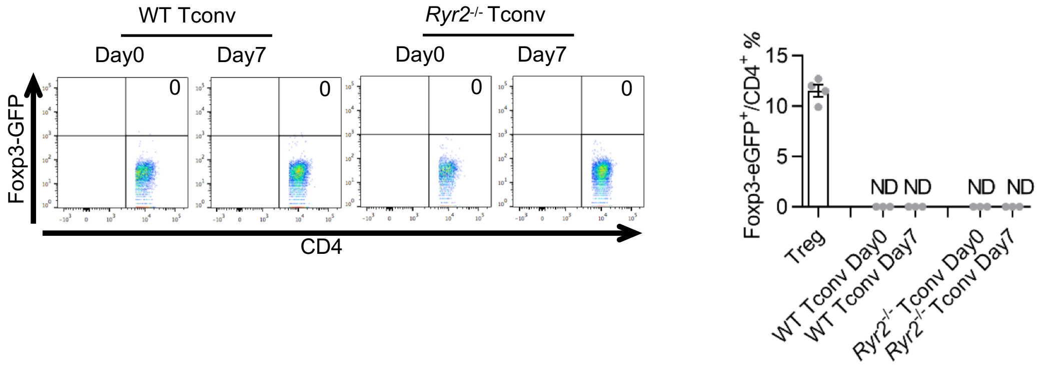

E

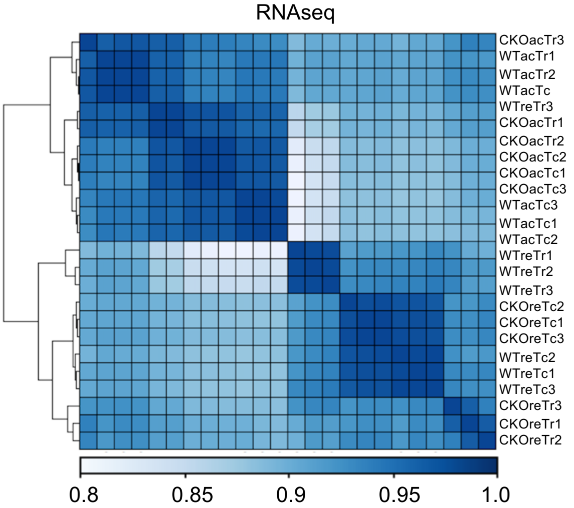

F

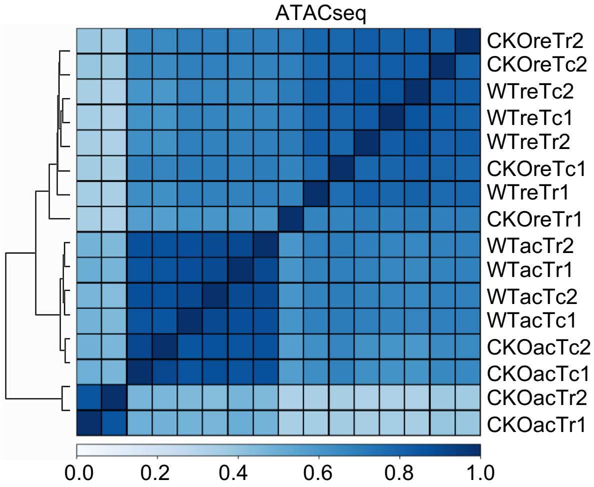

G

GeneName	WTreTc	CKOreTc	WTreTr	WTacTc	CKOacTc	WTacTr
Foxa1	0.00	0.04	0.33	0.00	0.00	0.02
Foxa2	0.00	0.00	0.03	0.00	0.00	0.00
Foxa3	0.00	0.00	0.04	0.04	0.16	0.09
Foxb1	0.00	0.00	0.00	0.51	0.02	0.14
Foxc1	0.00	0.01	0.04	0.05	0.05	0.13
Foxc2	0.11	0.10	0.06	0.00	0.00	0.00
Foxd1	0.00	0.00	0.00	0.00	0.00	0.00
Foxd2	0.72	0.49	0.96	0.50	0.25	0.62
Foxd2os	1.87	0.81	1.25	1.08	0.78	1.78
Foxd3	0.00	0.00	0.00	0.00	0.03	0.00
Foxd4	0.00	0.00	0.00	0.00	0.00	0.00
Foxe1	0.00	0.00	0.00	0.05	0.00	0.00
Foxf1	0.02	0.03	0.03	0.00	0.00	0.02
Foxf2	0.03	0.00	0.00	0.15	0.03	0.08
Foxh1	0.00	0.00	0.18	0.00	0.00	0.18
Foxi1	0.00	0.00	0.03	0.03	0.03	0.00
Foxi3	0.00	0.00	0.00	0.00	0.00	0.00
Foxj1	0.05	0.23	0.35	2.54	0.78	1.28
Foxj2	16.36	12.99	15.75	6.73	6.08	9.45
Foxj3	8.29	10.99	12.42	8.62	8.99	9.62
Foxk1	31.83	39.18	22.07	15.92	16.72	15.77
Foxk2	12.98	11.55	11.92	13.83	14.25	16.24
Foxl2	0.00	0.04	0.00	0.00	0.00	0.00
Foxl2os	0.00	0.02	0.00	0.04	0.00	0.05
Foxm1	2.41	2.21	2.50	2.23	1.33	4.16
Foxn1	0.00	0.00	0.00	0.00	0.00	0.02
Foxn2	18.86	15.86	15.91	16.00	16.13	11.96
Foxn3	17.46	18.74	16.34	11.62	11.97	13.44
Foxo1	25.96	32.56	29.51	16.58	14.61	17.43
Foxo3	15.16	18.25	16.18	10.69	11.33	13.23
Foxo4	17.51	27.64	25.38	6.23	5.38	10.87
Foxo6	0.00	0.05	0.00	0.00	0.00	0.00
Foxp1	20.14	29.17	18.10	22.64	24.43	19.06
Foxp2	0.01	0.00	0.00	0.01	0.00	0.02
Foxp3	0.62	2.83	118.29	1.07	1.96	123.45
Foxp4	22.00	22.78	27.00	24.49	27.40	29.55
Foxq1	0.04	0.30	0.66	0.15	0.04	0.01
Foxr1	0.00	0.00	0.00	0.00	0.00	0.00
Foxred1	7.08	5.98	6.41	7.03	8.89	6.83
Foxred2	2.89	2.28	2.24	5.21	4.84	6.05
Foxs1	0.14	0.00	0.00	0.17	0.00	0.00

H

GeneName	WTreTc	CKOreTc	WTreTr	WTacTc	CKOacTc	WTacTr
Ryr1	0.13	0.05	0.04	0.13	0.10	0.08
Ryr2	5.10	0.00	0.04	3.02	0.22	0.48
Ryr3	0.25	0.31	0.71	0.04	0.05	0.12
Cacna2d4	3.59	2.26	3.80	0.40	0.46	1.32
Ahnak	40.30	22.49	59.47	9.65	8.48	25.64
Itpr1	6.03	6.33	5.08	4.24	3.91	4.46
Itpr2	23.20	19.14	24.23	7.32	5.26	11.56
Itpr3	37.00	37.82	29.29	6.29	5.77	11.78
Stim1	13.87	18.14	11.79	5.93	6.19	7.33
Stim2	12.84	15.45	18.11	17.89	19.05	13.99
Trpm1	0.13	0.10	0.06	0.00	0.00	0.16
Trpm2	0.20	1.29	0.13	0.00	0.00	0.01
Trpm3	0.00	0.00	0.00	0.00	0.00	0.00
Trpm4	1.46	1.34	2.10	1.09	0.84	1.80
Trpv1	0.00	0.00	0.00	0.00	0.00	0.00
Trpv2	21.08	16.50	24.14	11.51	9.89	22.63
Trpv3	0.04	0.00	0.00	0.00	0.00	0.00
Trpv4	0.06	0.12	0.02	0.05	0.02	0.13

Supplemental Figure 4. RyR2 deficiency per se mediates contact-dependent suppression. (**A**) Analysis of surface markers potentially associated with Treg functions in *Ryr2*$^{+/+}$ and *Ryr2*$^{-/-}$ Tconvs by flow cytometry. N=3. (**B**) IL-10 and TGF-β from Treg, *Ryr2*$^{+/+}$ and *Ryr2*$^{-/-}$ Tconv cells after 72 hours anti-CD3 plus anti-CD28 stimulation were tested by ELISA. n=2 per group. N=3. (**C**) eGFP-*Ryr2*$^{-/-}$ Tconv were FACS sorted and stimulated with anti-CD3/anti-CD28 for 48 hours, and eGFP expression were analyzed. WT Tconv were analzyed as control. n=3-4 per group. N=3. (**D**) eGFP-*Ryr2*$^{-/-}$ Tconv were FACS sorted then transferred into syngeneic *Rag1*-KO mice. At day7, eGFP expression were analyzed in splenocytes. Donor cells (Day0) were analyzed as well. WT Tconv were analyzed as control. n=3-4 per group. N=3. (**E**) Spearman correlation and hierarchy clustering of the RNAseq data. WT or CKO Tconvs and Tregs were sequenced in resting (eg. WTreTc) or anti-CD3/CD28 activated (eg. WTacTc) status. Euclidean distance among RNAseq samples were shown. (**F**) Spearman correlation among ATACseq samples. (**G**) Transcription level of Forkhead family members. FPKM of each gene was shown. (**H**) Transcription level of T cell associated calcium regulators. FPKM of each gene was shown. ND or n.d., no detection.

Supplemental Figure 5

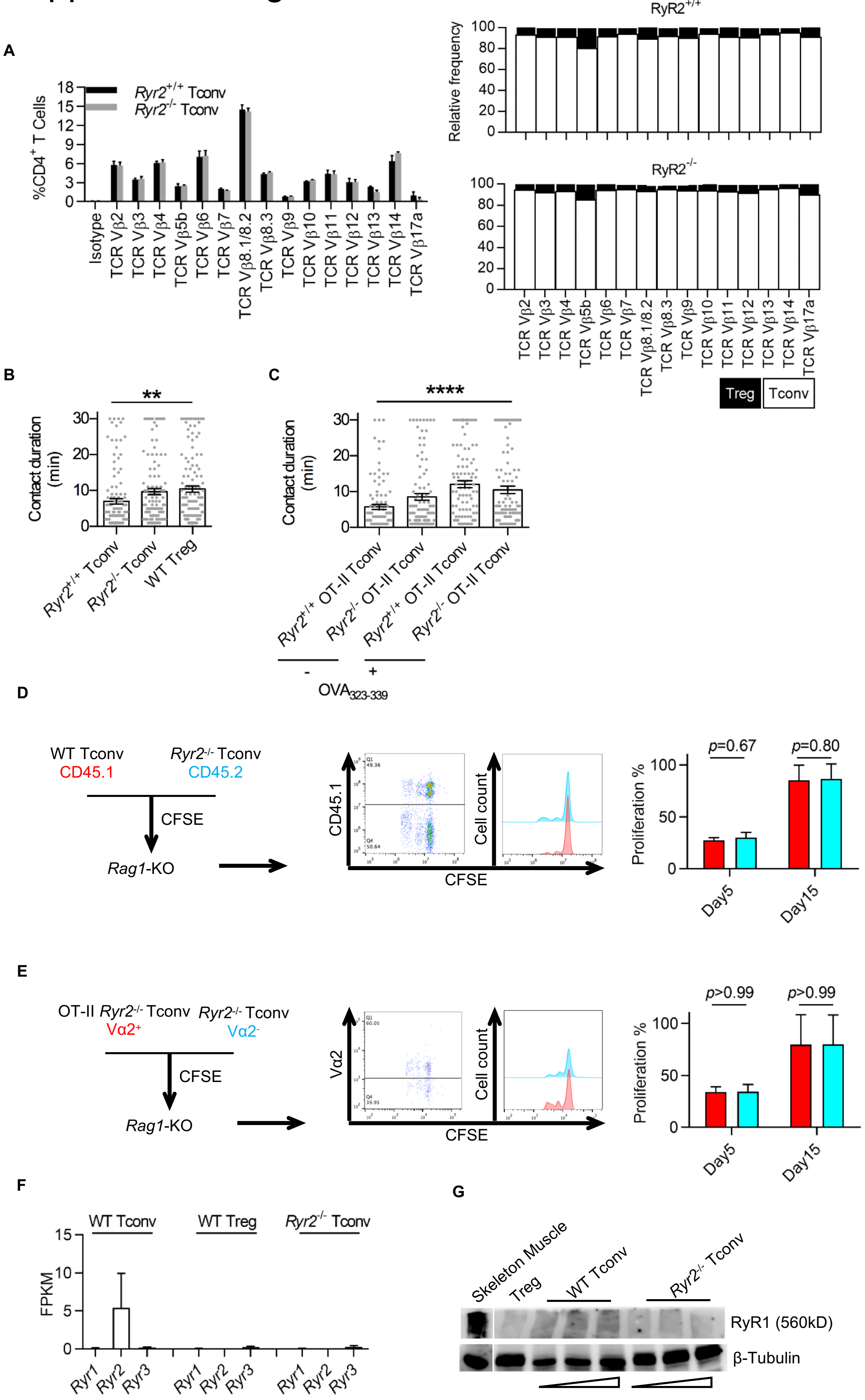

Supplemental Figure 5. RyR2 deficiency-mediated suppression operates in the absence of specific antigen. (**A**) T cell receptor Vβ chains of splenic $CD4^{+}$ T cells from CKO and WT mice were assessed. The expression of TCR vβ chains in splenic $CD4^{+}$ T cells for peripheral TCR usage were analyzed. n=3 per group, N=3. (**B**) Basal contact duration of *Ryr2*$^{+/+}$ Tconv, *Ryr2*$^{-/-}$ Tconv and Treg cells to DC *in vivo*. CD11c-DTR-eGFP transgenic mice were *i.v.* transferred with labeled *Ryr2*$^{+/+}$ Tconv, *Ryr2*$^{-/-}$ Tconv and WT Treg cells at 1:1:1 ratio. 120 contacts from 30~50 Tconv/Treg cells were analyzed. One-way ANOVA with nonparametric Kruskal-Wallis test. (**C**) Antigen-specific contact duration of *Ryr2*$^{+/+}$ OT-II Tconv, *Ryr2*$^{-/-}$ OT-II Tconv. CD11c-DTR/eGFP transgenic mice were i.v. transferred with labeled *Ryr2*$^{+/+}$ OT-II Tconv or *Ryr2*$^{-/-}$ OT-II Tconv, then inoculated with $OVA_{323\text{-}339}$ mixed with LPS at right abdomen region. Both draining inguinal lymph node ($OVA_{323\text{-}339}$+) and control node ($OVA_{323\text{-}339}$ free) were analyzed. 100 contacts from 20~50 cells are analyzed. Data are pooled from 3 independent experiments. One-way ANOVA with nonparametric Kruskal-Wallis test. (**D**) Mixed transfer of *Ryr2*$^{-/-}$ and wildtype Tconv (1:1) showed comparable proliferation in *Rag1*-KO recipient. CD45.1 B6 mice and CD45.2 *Ryr2*$^{-/-}$ mice were used. Cell proliferation were analyzed based on CFSE dilution at day 5 and day 15 after transfer. n=3 per group. 2-tailed unpaired Student's t test. (**E**) Cell proliferation between OT-II-*Ryr2*$^{-/-}$ Tconv and WT *Ryr2*$^{-/-}$ ones in *Rag1*-KO recipient were analyzed based on CFSE dilution at day 5 and day 15 after transfer. n=3 per group. 2-tailed unpaired Student's t test. (**F**) Plotting of RyR family transcription in WT Tconv, WT Treg and *Ryr2*$^{-/-}$ Tconv cells in RNAseq data in terms of FPKM. (**G**) RyR1 protein expression was detected in *Ryr2*$^{-/-}$ Tconv cells. Three increasing doses of loaded protein were analyzed and indicated in triangle.

Supplemental Figure 6

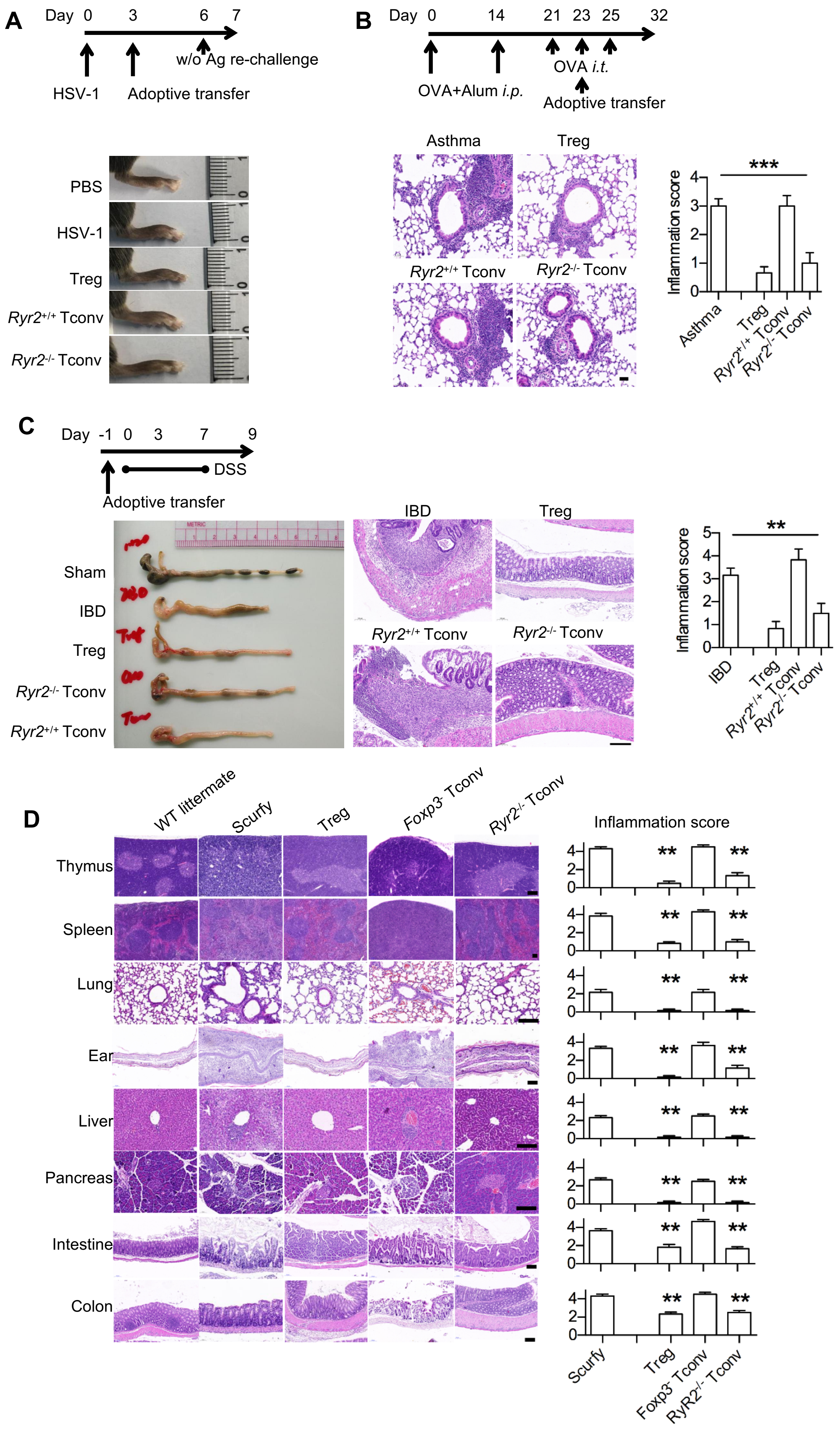

Supplemental Figure 6. RyR2-deficient Tconvs are indistinguishable from Tregs in disease models and scurfy rescue. (**A**) Experimental scheme (upper) and representative footpad swelling images in one DTH assay (lower left). (**B**) Sensitization scheme (upper) and representative lung H&E histological sections (lower). Scale bars represent 200 μm. Tissue inflammations were scored as methods stated. n=6 per group. One-way ANOVA with nonparametric Kruskal-Wallis test. (**C**) Induction scheme of DSS-induced colitis model (left), representative colon images (lower left) and photomicrograph of colon sections (lower right). Scale bars represent 200 μm. Tissue inflammations were scored as methods stated. n=6 per group. One-way ANOVA with nonparametric Kruskal-Wallis test. (**D**) Representative H&E staining of samples taken from indicated organs or anatomic sites. For WT and Scurfy and *Foxp3*$^{-}$ Tconv infused Scurfy, samples were taken on week 3. Rescued Scurfy mice were taken on week 8-12. Tissue inflammations were scored. n=6 per group. One-way ANOVA with nonparametric Kruskal-Wallis test.
